# Autobiographical memory in Alzheimer’s disease: a systematic review

**DOI:** 10.3389/fneur.2025.1546984

**Published:** 2025-06-16

**Authors:** Chiara Stramba-Badiale, Fabio Frisone, Diana Biondi, Giuseppe Riva

**Affiliations:** ^1^Applied Technology for Neuro-Psychology Laboratory, IRCCS Istituto Auxologico Italiano, Milan, Italy; ^2^Human Technology Laboratory, Università Cattolica del Sacro Cuore, Milan, Italy; ^3^Department of Psychology, Università Cattolica del Sacro Cuore, Milan, Italy; ^4^GARiP Recreational and Problem Gambling Laboratory, Università degli studi della Campania “Luigi Vanvitelli”, Caserta, Italy

**Keywords:** Alzheimer’s disease, autobiographical memory, self-defining memories, memory phenomenology, temporal gradients

## Abstract

**Introduction:**

Autobiographical memory impairment is a significant feature of Alzheimer’s disease (AD), affecting patients’ ability to recall personal life events and maintain their sense of self. While this impairment has been extensively studied, its aspects and manifestations remain incompletely synthesized in the literature regarding the relationship between memory specificity, temporal gradients, and emotional processing.

**Methods:**

We conducted a systematic review following PRISMA guidelines, searching across PubMed, Scopus, ScienceDirect, and Web of Science databases. Studies comparing autobiographical memory performance between AD patients and healthy controls were included. Quality assessment used Yang’s methodological checklist to evaluate potential bias in the selected studies. The review process involved independent analysis by two reviewers who assessed titles, abstracts, and full papers against predefined inclusion criteria.

**Results:**

Analysis of 83 studies revealed consistent autobiographical memory deficits in AD patients. These deficits were characterized by reduced memory specificity across all life periods, with patients showing a tendency toward overgeneralization. The studies demonstrated altered temporal gradients, with remote memories showing better preservation than recent ones, supporting Ribot’s law. Emotional processing patterns were also modified, with some studies indicating a positivity bias in memory recall. Various stimuli showed differential effectiveness in memory retrieval, with music and odors demonstrating particular promise compared to other cues. Neural correlates indicated involvement of hippocampal, prefrontal, and posterior cortical regions in autobiographical memory deficits. The research revealed significant correlations between autobiographical memory performance and executive function measures. Despite memory impairment, evidence suggested preserved components of self-reference.

**Discussion:**

The findings suggest that autobiographical memory impairment in AD affects multiple cognitive domains and impacts patients’ sense of self and quality of life. The identified patterns of impairment and preservation offer potential therapeutic targets and diagnostic markers. These results emphasize the need for standardized assessment protocols for autobiographical memory in AD and suggest the importance of developing targeted interventions leveraging preserved memory systems. The integration of multiple stimulus modalities in memory rehabilitation appears promising. The relationship between autobiographical memory and self-identity maintenance warrants further investigation. The review also highlights the importance of early detection and intervention in autobiographical memory deficits as potential markers of disease progression.

**Systematic Review Registration:**

https://www.crd.york.ac.uk/prospero/, identifier CRD42024596837.

## 1 Introduction

Alzheimer’s disease (AD), the most prevalent form of dementia, is a critical health condition responsible for an estimated 60–70% of all dementia cases globally (1). Much like coronary artery disease affects the heart, AD is a type of brain disease (2). The disease originates from the deterioration and destruction of brain cells, specifically neurons. It is characterized by its degenerative nature, progressively worsening over time (3). Notably, AD is believed to begin its insidious course 20 years or more before any symptoms become apparent, with initial brain changes occurring unnoticed by the affected individual (4). Only after years of these subtle alterations do noticeable symptoms, such as memory loss and language difficulties, manifest (5).

In the United States alone, AD affects over five million individuals aged 65 and above, ranking as the nation’s sixth leading cause of mortality (2). Current models of AD pathophysiology propose a temporal sequence of biological events. These theories suggest that the pathological process begins with disruptions in the production and/or clearance of the amyloid-beta (Aβ) protein (6). These disturbances trigger a cascade of biological events leading to the formation of amyloid plaques, which progressively spread throughout the cerebral cortex (3). Subsequently, pathological accumulation of tau protein (tauopathy) occurs, followed by neuronal dysfunction and death, ultimately culminating in the clinical manifestation of dementia (6). Advanced imaging techniques, such as Positron Emission Tomography (PET) and Magnetic Resonance Imaging (MRI), play a crucial role in assessing these pathological processes (7). These techniques allow for precise quantification and localization of various disease markers, including the distribution of amyloid-beta plaques, the extent of tauopathy, changes in cerebral glucose metabolism, and structural decline of brain tissue.

Since the progressive neurodegenerative disorder impacts the memory and various cognitive functions, as well as social interactions, emotional processing, and the sense of self, it is impaired (8). Research has established that memory performance in AD patients differs markedly from that of Healthy Controls (HC) (9), with some studies suggesting that memory impairment is among the earliest detectable manifestations of AD (10).

This deterioration extends across multiple memory systems, including autobiographical memory (AM), which encompasses the ability to recall personal life events that are essential for maintaining a stable sense of self (11, 12).

AM can be categorized into semantic memory, which encompasses self-related knowledge, and episodic memory, which involves detailed recollections of personal past events (13). The episodic component is regarded as the hallmark of autobiographical memory recall, enabling individuals to vividly reconstruct past experiences (14, 15).

Several studies have highlighted different key features that contribute to autobiographical memories’ phenomenology and that are relevant to understanding AM decline in AD. These include: (a) vividness, defined as the amount of perceptual or sensory details recalled (16); (b) belief in the accuracy of memories, though it can be influenced by factors such as emotional valence and narrative coherence (15); (c) sensory details, including visual, auditory, and tactile information, that are crucial in enhancing the recollection process during memory retrieval (14); (d) emotional valence and intensity, with positive emotions often aiding in better encoding and retrieval of memories (17). Then, the accessibility and frequency of sharing autobiographical memories have been recognized as important dimensions (18).

Recent research has also highlighted the importance of narrative coherence in understanding individual differences in memory experiences (19). The progressive deterioration of AM in AD has profound and pervasive consequences. This decline is not limited to the mere loss of memories but extends to the gradual erosion of knowledge about events and facts that have shaped the patient’s life. Consequently, there is an inexorable degradation of self-awareness and sense of personal identity (11). This process not only alters the patient’s perception of their past but also significantly influences their ability to situate themselves in the present and project into the future, thus compromising the essence of personal identity.

AD patients face significant challenges in recalling specific episodic details from their past. This difficulty in producing precise memories of individual events is a hallmark of the autobiographical compromise observed in AD (20–22).

Despite the significant decline, some aspects of AM may be relatively preserved in early AD. Semantic autobiographical knowledge, or general facts about one’s life, may be retained longer than specific episodic memories (23). Additionally, the emotional content of memories may be preserved to some extent (11).

The relationship between AD patients’ subjective experience of remembering and their actual recall ability presents an intriguing area of study. This discrepancy can be better understood when considered alongside research on anosognosia in AD (24). Studies have revealed a notable mismatch between how AD patients evaluate their memory capabilities and their actual performance on memory tasks. This phenomenon highlights the complex nature of memory awareness in AD, where patients may not fully recognize or appreciate the extent of their memory distortions (25).

AM is distinguished not only by the vivid re-experiencing of past events, but also by its unique temporal distribution pattern. This pattern comprises three key phenomena: childhood amnesia, the reminiscence bump, and the recency effect (26–28).

Childhood amnesia refers to the near-total absence of memories from the earliest years of life. The reminiscence bump, in contrast, describes a substantial increase in memories for events that occurred between the ages of 10 and 30. Lastly, the recency effect reflects a tendency to more easily recall recent events (26–28).

Among these three phenomena, the reminiscence bump has garnered the most research attention, as it encompasses the most significant events in a person’s life. This bump is thought to result from numerous first-time experiences, which later serve as reference points when individuals encounter similar situations (29). The reminiscence bump represents the most crucial component for self-definition, comprising vivid and emotionally charged memories that profoundly impact one’s sense of self (19).

Neuroanatomically, AM decline in AD is associated with disruption of the default mode network (DMN), hippocampal and medial temporal lobe atrophy, and prefrontal cortex dysfunction (30, 31). These neuroanatomical changes align with current models of AD pathophysiology (6). Additionally, the shift from episodic to semantic memory content may be linked to compensatory overactivation of the left prefrontal cortex, suggesting the brain’s attempt to adapt to episodic memory loss.

The decline of AM in AD is a complex phenomenon with far-reaching impacts on patients’ sense of self and quality of life. Understanding the nuances of this decline is crucial for developing targeted interventions to preserve AM function and maintain the sense of self in AD patients.

Despite the importance of this topic, to date, no recent systematic review has been conducted to comprehensively synthesize the available evidence in this field. Therefore, the present study aims to address this gap in the scientific literature investigating the AM in patients with AD.

## 2 Methods

To outline the current state of the art about AM in AD, a literature search and analysis were conducted according to the preferred reporting items for systematic reviews and meta-analysis (PRISMA) guidelines (see the Supplementary Table 1) (32). Data sources were collected on May 24. The search was performed considering four databases: PubMed, Scopus, ScienceDirect, and Web of Science. The strings of words used to perform the search were those resulting from the combination of Alzheimer’s and the words generally used when referring to the AM (i.e., Autobiographical Memory, self-defining memory, etc.) (see Table 1) (33). The search was conducted according to the abstract, title, and keywords. A specific starting year of publication for the articles to be included was not defined beforehand, as we aimed to include all articles published to date that addressed this topic. Citations were retrieved from each search in each database and imported into Rayyan (34) to remove duplicates and to start the first screening.

**Table 1:** The table shows the characteristics of the studies that refer only to the research object of this review.

	PubMed	ScienceDirect	Scopus	Web of science
	Autobiographical memor* 267	Autobiographical memor 56	Autobiographical memor* 378	Autobiographical memor* 534
Alzheimer	Self-defining memor* 7	Self-defining memor 302	Self-defining memor* 9	Self-defining memor* 14
	Memor* phenomenology 25	Memor phenomenology 22	Memor* phenomenology 60	Memor* phenomenology 45
				
	1987 hits			

### 2.1 Selection criteria

Only studies that met the following inclusion criteria were included in this systematic review: (a) studies that directly compare an AD subsample to a healthy control group; (b) published in English; (c) contained quantitative and qualitative data concerning cognitive abilities (no case studies); (d) peer-reviewed publications (excluding the entire grey literature); (e) empirical studies (no reviews, no conceptual papers etc.); (f) samples with a diagnosis of AD or probable AD.

### 2.2 Quality assessment and data extraction

To control the risk of bias and ensure a fair review process, the PRISMA recommendations for conducting systematic reviews were strictly followed. The review was pre-registered in the PROSPERO register (CRD42024596837). The methodological quality of included studies was assessed using Yang et al.’s (35) checklist for comparative diagnostic accuracy studies. This tool was selected because it specifically addresses the unique challenges of evaluating studies that compare multiple assessment approaches, which is critical in our review, where various autobiographical memory measures were being compared across different populations. The QUADAS-C tool, as developed by Yang and colleagues, extends the widely used QUADAS-2 framework while maintaining its established four-domain structure (Patient Selection, Index Test, Reference Standard, and Flow and Timing). For our specific review question examining AM in AD, this tool provided several advantages over traditional quality assessment instruments. First, it allowed us to evaluate whether studies appropriately handled the comparison between patient and control groups. Second, it assessed whether the administration and interpretation of memory tests were conducted with appropriate blinding. Third, it evaluated whether the reference standard (diagnostic criteria for AD) was consistently applied. Finally, it examined whether patient flow and timing issues might have introduced bias in the comparison of AM performance. The checklist comprises four specific bias assessment criteria: (1) Could the selection of patients have introduced bias? (2) Could the conduct or interpretation of the index test have introduced bias? (3) Could the reference standard, its conduct, or its interpretation have introduced bias? and (4) Could the patient flow have introduced bias? Each study was evaluated against these criteria as detailed in Supplementary Table 1. Disagreements between reviewers (C.S.B., F.F.) were resolved through consensus discussion, with a third reviewer (D.B.) consulted when necessary.

### 2.3 Classification criteria

To address the heterogeneity in our systematic review and enhance the transparency of our analysis, we have categorized the included studies according to their methodological approaches and disease severity classifications. The diversity in study designs, assessment tools, and participant characteristics presents significant challenges when interpreting results across the literature on AM in AD.

After analyzing the selected studies, we proposed a narrative synthesis divided into eight main categories, as the impairment of AM in AD affects various aspects of cognitive functioning and quality of life (36–38).

The main themes included: (a) general AM deficits; (b) specificity of autobiographical memories; (c) temporal gradient of memories; (d) emotional components of AM; (e) effects of different stimuli on memory retrieval; (f) relationship between AM and sense of self, (g) comparison with other neurological disorders; and (h) neural correlates of AM in AD.

Given that some studies have shown overlapping deficits across different aspects of AM in AD (21), these have been included across multiple sections.

### 2.4 Search results

**Figure 1:**
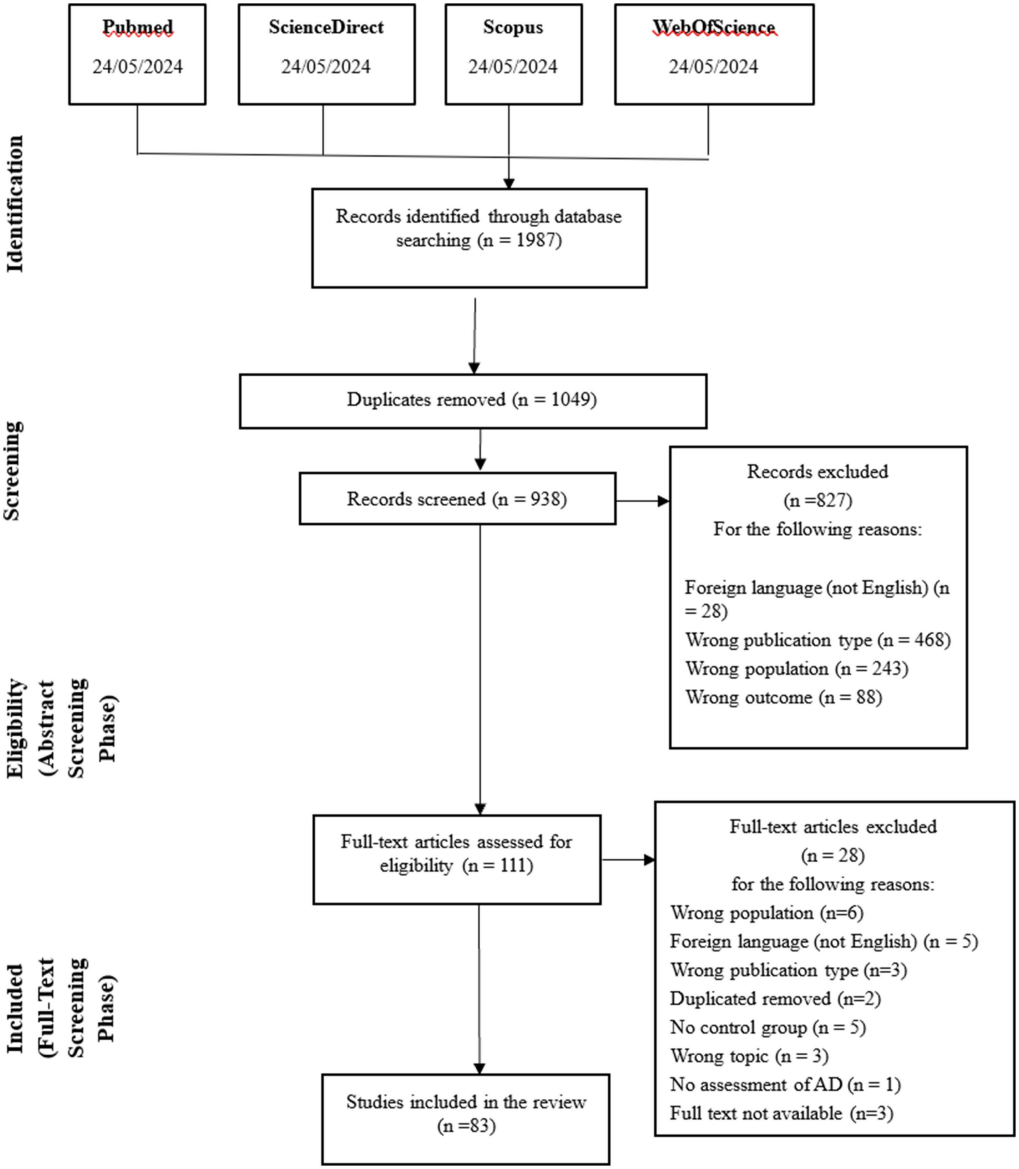
PRISMA-flowchart of the inclusion process (6).

The initial search yielded 1987 papers, of which 1,049 were duplicates and therefore removed from the list. A total of 938 records were obtained. The flow chart of the search strategy is shown in Figure 1, with details of the reasons for exclusion.

A total of 83 papers were included. Supplementary Table 2 shows, in alphabetical order, a summary of the studies that were selected based on the eligibility criteria. The purpose, the sample features, the measures and tasks for testing AM, and the main outcomes of each study were shown in the table (See Supplementary Table 2).

## 3 Results

### 3.1 Study methodology

**Figure 2:**
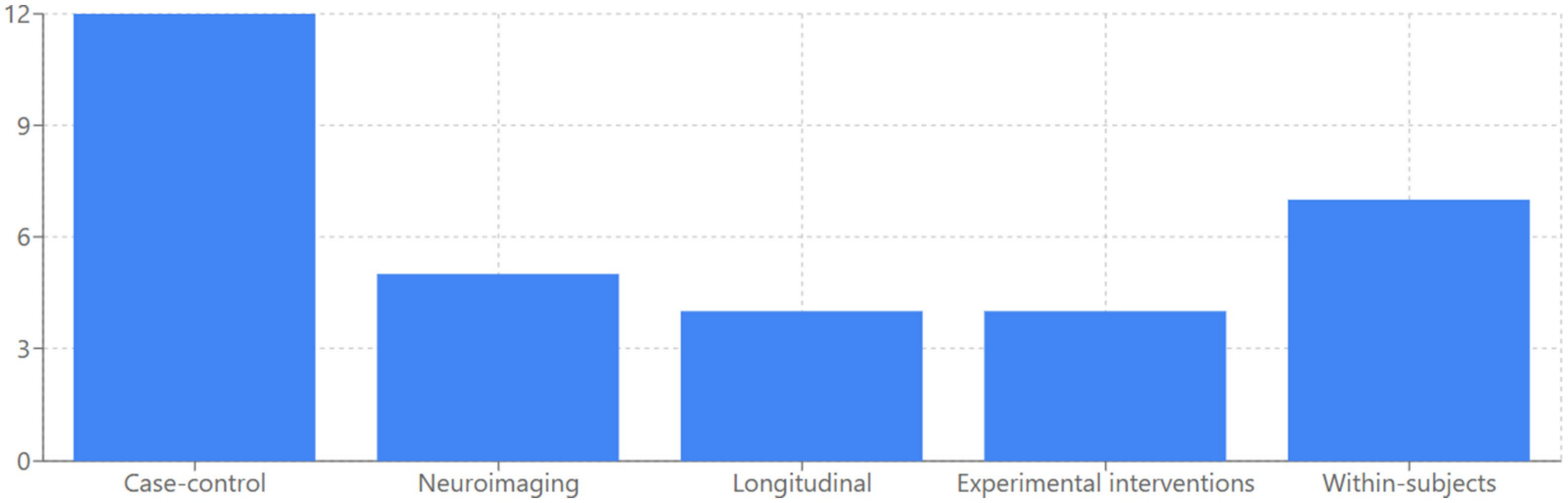
Study methodologies used in AD research.

The heterogeneity in study design across the reviewed literature can be categorized into five main methodological approaches. Cross-sectional case–control studies constituted the predominant approach (23, 37, 39–49), allowing for direct comparison between AD patients and healthy controls at a single time point. Correlational neuroimaging studies (50–54) investigated the relationship between AM performance and neural substrates using various imaging techniques. Longitudinal designs were less common but provided valuable insights into memory deterioration over time (36, 55–57). Experimental interventions were implemented in several studies (11, 49, 58, 59) to test specific memory enhancement techniques. Finally, within-subjects experimental designs (22, 60–65) examined how different conditions (e.g., music, odors) affected AM retrieval in the same participants, providing more controlled comparisons while minimizing the influence of individual differences. The distribution of studies based on methodology is represented in Figure 2.

### 3.2 Autobiographical memory assessment

**Figure 3:**
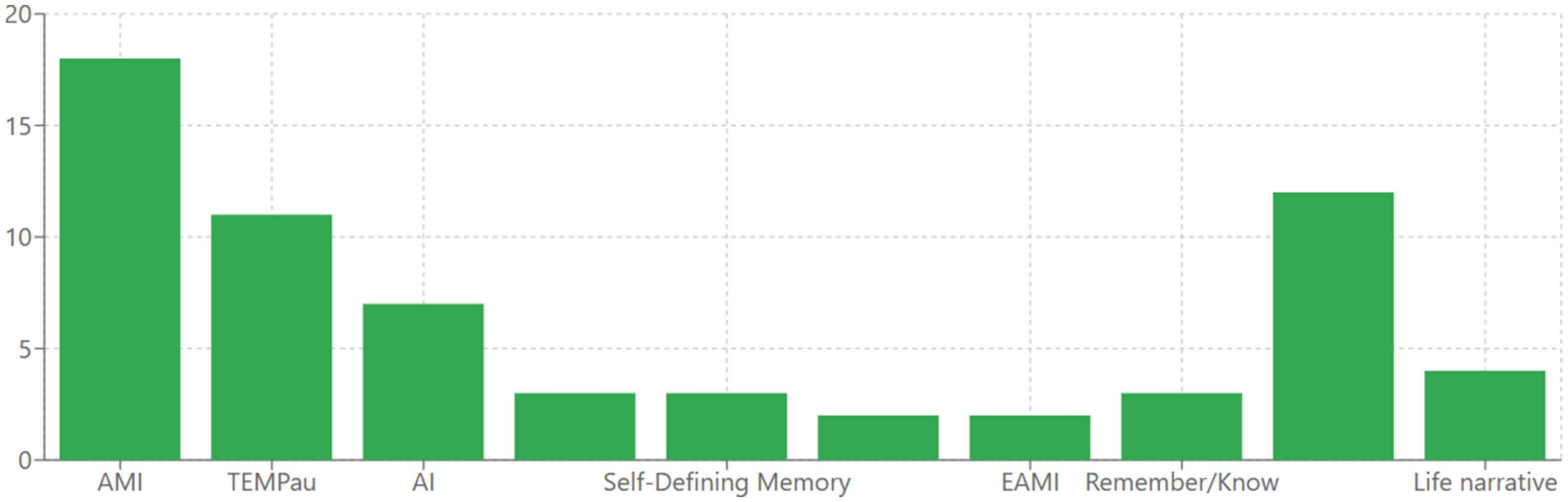
Autobiographical memory assessment tools used in AD research.

The studies examining AM in AD employed a variety of assessment tools, revealing methodological heterogeneity that warrants careful interpretation. The most frequently used instrument was the Autobiographical Memory Interview (AMI), implemented in numerous studies (21, 36, 39, 41, 42, 52, 60, 66–76). The Test Episodique de Mémoire du Passé (TEMPau) was another widely used instrument (22, 58, 59, 61, 63, 64, 77–81). Several researchers employed the Autobiographical Interview (AI) or adapted versions of it (21, 37, 40, 44, 82–85). The Autobiographical Memory Test (AMT) was used in studies focusing on memory specificity (43, 46, 86). Other assessment tools included the Self-Defining Memory Task (SDM) (87) in studies examining identity and self-concept (45, 88, 89), the Extended Autobiographical Memory Inventory was used by Hirjak (54) and Seidl (90) and the Episodic Autobiographical Memory Interview (EAMI) implemented by Rodrigues (91) and Glachet (62). Several studies used the Remember/Know paradigm to assess autonoetic consciousness (53, 78, 92). More specialized approaches included music-evoked and odor-evoked autobiographical memories (22, 57, 60–64, 79, 93–95, 96) and life narrative methods (47, 48, 96, 97). The various AM assessment tools used across studies are visually presented in Figure 3.

### 3.3 Disease severity progression

**Figure 4:**
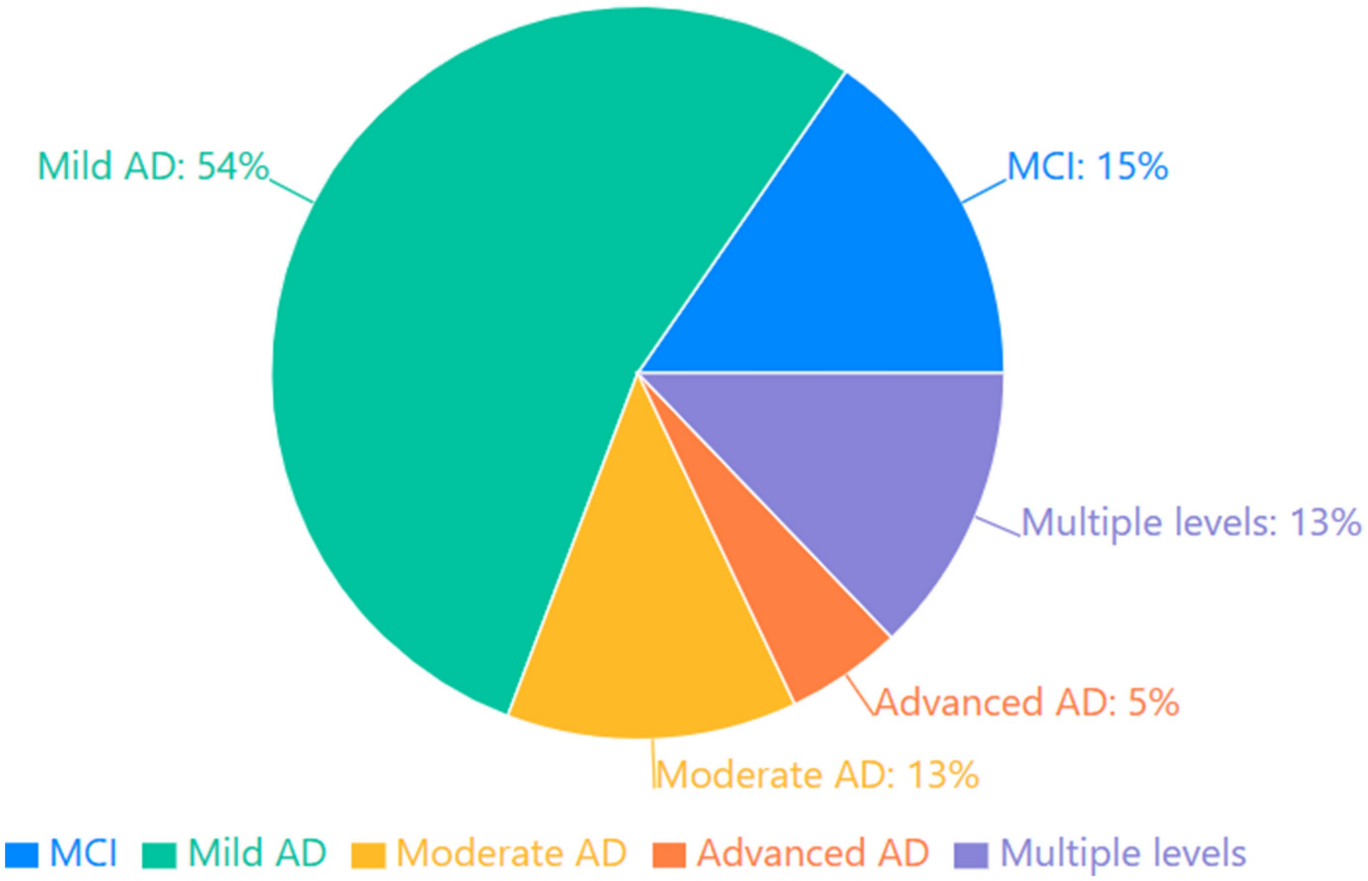
Distribution of AD severity in studies.

The reviewed studies encompassed a spectrum of disease severity, which can be categorized into four distinct stages of progression. Studies focusing on preclinical and prodromal stages included participants with mild cognitive impairment (MCI) and those at high risk for developing AD (21, 47, 50, 54, 86, 90). Mild AD was the most common severity level studied (Mini Mental State Examination- MMSE scores typically 21–26), featured in numerous investigations (11, 20, 22, 38, 49, 58–60, 62, 77, 79, 89, 98–103). Moderate AD studies (MMSE scores 16–20) provided insights into more advanced memory deterioration (5, 49, 61, 74, 93, 96). Several studies included participants with severe AD (MMSE scores below 16), revealing patterns of severe autobiographical memory loss (82, 104). Importantly, some investigations spanned multiple severity levels, enabling examination of the progression of AM impairment across the disease continuum (46, 68, 69, 72, 105). The Distribution of AD Severity in Studies is shown in Figure 4.

### 3.4 General deficits of autobiographical memory in Alzheimer’s disease

With the progression of AD, there’s a general decline in AM, but the rate and pattern of this decline can vary across different components of AM (21, 36–43, 55, 56, 66–71, 86, 90, 91, 96–99, 104–106).

This decline manifests in multiple dimensions of AM functioning. Firstly, AD patients exhibit consistent and significant impairments in AM performance relative to HC across diverse empirical investigations (36, 38, 44, 60, 100). Secondly, deficits are observed in both the episodic and semantic components of AM, with a disproportionate impairment typically evident in the episodic domain (67, 90, 100). AM deficits are detectable in the prodromal and early stages of AD and demonstrate a progressive deterioration that correlates with disease advancement (39, 68).

Moreover, it was demonstrated that HC could selectively inhibit episodic AM, while patients with mild AD showed impaired intentional inhibitory processes for both episodic and semantic AM, suggesting that inhibitory deficits may precede general AM deterioration in AD (100). Over 18 months, the episodic memory of HC patients remained stable, with no signs of decline, while the amnestic mild cognitive impairment (aMCI) group manifested a less pronounced decline in episodic memory compared to AD (36).

Furthermore, studies have also highlighted the complexity of AM impairment in AD, including deficits in specificity (37, 40, 45–48, 58, 59, 82, 88, 100, 101, 107, 108), emotional content (37, 46, 50, 61, 77, 83, 102), and temporal distribution of memories (38–40, 51, 52, 56, 68, 69, 78, 90, 109).

### 3.5 The specificity of autobiographical memory in Alzheimer’s disease

AD patients consistently produce less specific and more generalized AMs compared to HC (37, 40, 43, 100, 107). This pattern is reflected in AM narratives, with the majority of the studies reporting a reduction in internal (episodic) details and an increase in external (semantic) details in AD patients (37, 38, 84).

While this pattern is generally consistent, some variations have been observed. For instance, Strikwerda-Brown et al. (108) found that AD patients provided significantly more specific episode external details compared to HC. Notably, the loss of specificity is not uniform across the lifespan, as it affects memories from different life periods but is often more pronounced for recent memories (37, 82). Patients with semantic dementia (SD) exhibited a relative preservation of recent AMs compared to memories from more distant periods. In contrast, individuals with behavioral variant frontotemporal dementia (bvFTD) and AD demonstrated a consistent impairment in recalling specific and detailed AMs across all life periods, regardless of whether they were asked to recall freely or were provided with prompts.

### 3.6 Emotional components of autobiographical memory in Alzheimer’s disease

The emotional valence of AMs in AD patients showed mixed patterns across studies. Some research suggested that AD patients exhibit a positivity bias, recalling more positive than negative memories compared to HC (20, 61, 77). However, contrasting findings by Meléndez et al. (46) indicated a tendency toward emotional neutrality, with AD patients showing fewer positive and negative memories but more neutral ones compared to HC and MCI groups. Despite these variations in emotional valence, the emotional intensity of memories is sometimes preserved in AD, even with reduced specificity (50, 98).

Overall, emotional regulation in AM recall appears altered in AD, with patients demonstrating different patterns of emotional processing compared to HC (37, 83).

### 3.7 Temporal gradient of autobiographical memories in Alzheimer’s disease

Many studies reported a temporal gradient in AD, with better preservation of remote memories compared to recent ones (51, 52, 56, 68, 69). Recent memories were typically more impaired, with significant deficits observed for events from the past few years (23, 39, 51, 90, 109). However, this pattern is not universal. Addis et al. (38) found deficits in both recent and remote AMs, while Rauchs et al. (78) observed relatively better recall for very recent events (today/yesterday) compared to intermediate periods.

Interestingly, the reminiscence bump (enhanced recall for events from young adulthood) is often preserved in AD, although these memories may be less specific than in HC (66, 97).

These findings highlight the complexity of AM impairment in AD and suggest that factors beyond mere temporal distance may influence memory preservation and retrieval.

Additionally, research has shown that different types of cues can differentially affect AM retrieval in AD patients (22, 49, 57, 60–65, 79, 80, 93, 94, 103, 110), suggesting that the method of memory elicitation is crucial in assessing AM deficits.

Studies have also demonstrated the relationship between AM deficits in AD and other neurological disorders (41, 67, 72, 84, 85, 92), as well as changes in the sense of self (20, 45, 53, 73, 74, 81, 89, 111–113), emphasizing the importance of a comprehensive approach to understanding AM in this condition.

The fluctuating nature of AM impairment across different stages of AD (21, 37, 43, 55, 56, 70, 71, 86, 91, 97, 98, 105, 106) also suggests that a classification based solely on disease severity may not capture the full complexity of AM deficits.

### 3.8 Effects of different stimuli on autobiographical memory retrieval in Alzheimer’s disease

Various studies have examined the effectiveness of different stimuli in AM retrieval among AD patients. Regarding visual stimuli, Kirk et al. (49) demonstrated that concrete objects were more effective than words, while Lopis et al. (94) found that pictures outperformed olfactory stimuli. Studies by Glachet et al. (62, 64, 65) revealed that odors significantly improved memory retrieval and access to self-concept in AD patients. Music proved particularly effective, as evidenced by research from Irish et al. (60), Baird et al. (57, 95), and Cuddy et al. (93). El Haj et al. (22) conducted a series of in-depth studies on music: in 2012, they discovered that participant-chosen music evoked more specific memories; in 2013, they observed linguistic improvements in AD patients’ autobiographical narratives during music listening (63); in 2015 (79), they showed that self-chosen music produced more self-defining memories than researcher-chosen music or silence. In 2018, they compared olfactory and musical cues (80), while in 2020, they demonstrated the benefits of using pictures alongside verbal instructions (103). Baird et al. compared the effectiveness of music and photographs (57, 95), while Rasmussen et al. (110) used films, observing an increase in involuntary memories and emotional reactions in AD patients.

### 3.9 Relationship between autobiographical memory and the sense of self in Alzheimer’s disease

Research on the relationship between the sense of self and AM in AD revealed a complex picture (53, 74, 89, 111). AD patients have difficulties retrieving specific AMs and episodic details compared to healthy older adults, but personal semantic memories tend to be better preserved (45, 73). Despite these memory impairments, research suggested that AD patients in mild to moderate stages can still retrieve some self-defining memories that contribute to their sense of identity (45, 73, 79).

Interestingly, El Haj et al. (112) found that AD patients demonstrated an ability to reflect on their sense of self-continuity by retrieving AMs, particularly when wanting to feel they are the same person as before (81).

The emotional valence and integration of self-defining memories also appear relatively intact in mild AD compared to the healthy group (61). Furthermore, AD patients rate their self-defining memories as central to their identity and contributing to self-continuity at similar levels to healthy older adults (111).

### 3.10 Comparison with other neurological disorders

AM impairments in AD show both similarities and differences compared to other neurological conditions. For instance, AD patients showed impaired AM but relatively preserved semantic memory, while SD patients exhibited severely impaired semantic memory but relatively preserved AM (67, 92).

Moreover, SD patients often show a reversed temporal gradient compared to AD, with better preservation of recent memories (23, 67, 72, 85).

Patients with bvFTD may show different patterns of AM impairment compared to AD, particularly in emotional content and self-reference (37).

Logopenic Progressive Aphasia (LPA) patients demonstrated widespread deficits in spontaneous recollection across all temporal epochs, with their performance matching that of AD patients at comparable stages of illness progression.

The pattern of AM deficits can help differentiate AD from other forms of dementia and MCI (41).

### 3.11 Neural correlates of autobiographical memory in Alzheimer’s disease

Neuroimaging studies have identified several brain regions associated with AM deficits in AD. For instance, hippocampal atrophy is consistently linked to impaired episodic AM (23, 39, 40, 51, 52, 54, 75, 114). Moreover, prefrontal cortex dysfunction is associated with reduced specificity and organization of AMs (40, 44, 52, 75). Posterior cortical regions, including the precuneus and retrosplenial cortex, are implicated in the retrieval of AMs (75, 78, 85, 115). Neural correlates common to both recent and remote periods were identified, including the hippocampus, medial prefrontal, and frontopolar cortices, and the forceps minor and left hippocampal portion of the cingulum bundle (40, 56, 76, 85). The neural substrates of AM in AD show both commonalities and differences compared to healthy aging, reflecting both normal age-related changes and pathological processes (40, 75). Additionally, disrupted connectivity between the hippocampus and prefrontal regions, and between temporal and frontal networks has been observed, with compensatory activation patterns in the left inferior frontal gyrus, ventromedial prefrontal cortex, and left lingual gyrus (85). These neural changes correlate with episodic memory performance, semantic memory deficits, executive function measures, and general cognitive decline, following a progression pattern that initially involves the hippocampus, then fronto-temporal networks, and finally posterior cortical regions (50, 52, 78, 85).

## 4 Discussion

The results of the selected studies, examining AM in AD, as summarized in Supplementary Table 2, revealed significant differences between AD patients and controls, with important clinical implications.

Observing the general deficits of AM in AD, it is clear that AM plays a crucial role in disease progression. This may reveal a broad cognitive decline and distinct patterns across episodic and semantic memory components, which could serve as early diagnostic markers and targets for intervention. Regarding the specificity of AM in AD, the findings reveal a consistent pattern of reduced specificity and increased generalization in AM narratives compared to HC.

A notable temporal pattern emerges, as most studies indicate that AD patients better preserve remote memories compared to recent ones, maintaining a “reminiscence bump” for young adulthood events. However, this pattern shows variations, suggesting that memory preservation is influenced by factors beyond mere temporal distance. Interestingly, research demonstrates that various stimuli can enhance AM retrieval, with self-chosen music, concrete objects, and odors proving particularly effective compared to traditional verbal cues.

Despite these impairments in retrieving specific AM, AD patients in mild to moderate stages maintain some ability to access self-defining memories and demonstrate a preserved sense of self-continuity. From an emotional perspective, findings highlight mixed patterns of emotional valence that could have significant implications for understanding how emotional regulation is altered in this population, suggesting the need for nuanced therapeutic approaches.

The distinctiveness of AM impairment in AD becomes particularly evident when compared to other neurological conditions, with AD patients showing unique characteristics compared to SD, bvFTD, and LPA. These insights not only enhance our comprehension of cognitive decline in AD but also highlight potential differences in memory preservation across various dementia types, which can inform targeted therapeutic approaches and diagnostic criteria.

Neuroimaging studies further illuminate this understanding, revealing that AM deficits in AD are associated with widespread brain changes, particularly in the hippocampus, prefrontal cortex, and posterior cortical regions. These changes follow a specific progression pattern from hippocampal to posterior cortical involvement, with disrupted connectivity patterns that provide a neural basis for the observed memory impairments.

When interpreting these findings, it is essential to consider the methodological approaches employed across literature. The heterogeneity in study design presents both challenges for synthesis and opportunities for comprehensive understanding. Cross-sectional case–control studies constituted the predominant approach (23, 37, 39–49) allowing for direct comparison between AD patients and HC at a single time point. This methodology provides valuable snapshots of cognitive differences but cannot capture the dynamic progression of autobiographical memory decline. Correlational neuroimaging studies (50–54) investigated the relationship between AM performance and neural substrates using various imaging techniques, offering critical insights into the neurobiological underpinnings of memory impairment. These studies have been instrumental in establishing connections between structural changes, particularly in hippocampal regions, and specific patterns of AM deficits. Longitudinal designs, though less common in the reviewed literature (36, 55–57), provided valuable insights into memory deterioration over time. These studies are particularly valuable as they allow tracking the trajectory of AM decline across disease progression, potentially identifying crucial transition points where intervention might be most effective. Experimental interventions implemented in several studies (11, 49, 58, 59) tested specific memory enhancement techniques, offering promising directions for clinical applications. Within-subjects experimental designs (22, 60–65) examined how different conditions (e.g., music, odors) affected AM retrieval in the same participants, providing more controlled comparisons while minimizing the influence of individual differences. These designs are particularly valuable for identifying personalized approaches to memory support that might be effective across different stages of the disease.

Moreover, literature reveals a substantial variety of assessment instruments, reflecting the multifaceted nature of AM and researchers’ evolving methodological approaches. Among these tools, AMI was most commonly employed (21, 36, 39, 41, 42, 52, 60, 66–76), likely due to its comprehensive evaluation of both personal factual knowledge and specific event recollection across the lifespan. This approach facilitates examination of memory changes through different life stages and temporal patterns. Researchers frequently utilized the TEMPau (22, 58, 59, 61, 63, 64, 77–81) when investigating experiential and conscious aspects of memory retrieval. The AI methodology and its variations (21, 37, 40, 44, 82–85) proved valuable for quantifying the balance between episodic details and semantic elaboration in personal narratives, highlighting the tendency toward overgeneralization in AD. For investigations specifically targeting memory specificity, the AMT (43, 46, 86) offered a structured approach to measure generalization patterns. Identity-focused research often employed the SDM (45, 88, 89), while specialized protocols for Extended Autobiographical Memory (54, 90) and the EAMI (62, 91) addressed different aspects of recollection. The subjective experience of remembering was evaluated through the Remember/Know paradigm (53, 78, 92) in several studies. Innovative approaches using sensory stimuli to evoke memories—particularly through music (22, 57, 63, 79, 80, 93, 95) and odors (60–62, 64, 94)—and naturalistic life story methods (47, 48, 96, 97) contributed unique perspectives on memory accessibility and content in AD. This methodological diversity reflects the multifaceted nature of AM and highlights the need for comprehensive assessment protocols that capture both quantitative deficits and qualitative changes in memory experience. Future research would benefit from incorporating multiple assessment approaches within single studies to allow for direct comparison of different memory components and to better characterize the complex pattern of preservation and impairment in AD.

Understanding the trajectory of autobiographical memory impairment requires examining performance across the continuum of AD severity. Our review identified distinct patterns of investigation across the disease spectrum. At the earliest stage, researchers examined prodromal manifestations in participants with MCI and those with genetic or biomarker risk factors for developing AD (21, 47, 50, 54, 86, 90). These studies have proven crucial for identifying autobiographical memory changes that may precede clinical diagnosis, potentially serving as early cognitive markers. Indeed, comparisons between AD patients, individuals with MCI, and controls provide valuable insights into the trajectory of AM decline. Research suggests that while MCI patients often show intermediate levels of impairment, their AM performance is generally closer to that of controls than AD patients, particularly for recent memories (39, 86). This pattern of decline could potentially aid in early detection and differentiation between normal aging, MCI, and AD. The majority of research concentrated on mild AD (11, 20, 22, 38, 49, 58–60, 62, 77, 79, 89, 98–103), capturing AM function at a stage where impairment is clinically significant, yet patients retain sufficient cognitive resources to meaningfully engage with complex assessment protocols. Research on moderate AD (5, 49, 61, 74, 93, 96) documented more pronounced deficits, while investigations into severe AD (82, 104) revealed the most pronounced patterns of AM deterioration. Particularly informative were the cross-sectional studies spanning multiple severity levels (46, 68, 69, 72, 105), which illuminated how different components of AM (semantic knowledge versus episodic detail, recent versus remote events) deteriorate at varying rates and patterns as the disease advances. This staged approach to examining AM across disease progression reveals not merely quantitative decline but qualitative shifts in memory characteristics and strategies for retrieval, with implications for both theoretical models of memory systems and practical approaches to cognitive support. The staging of disease severity across studies raises important considerations for clinical practice. The identification of AM declines in preclinical and early stages suggests potential value as a diagnostic marker. However, the heterogeneity in assessment approaches and the variability in how disease severity was operationalized across studies highlight the need for standardization in both assessment protocols and severity classification to enhance comparability across research and clinical settings.

While staging approaches illuminate the temporal evolution of AM deterioration across disease progression, comparative analyses provide complementary insights into the qualitative nature of these deficits. Bridging these perspectives enhances our understanding of both the progression and manifestation of memory impairments. Consistently through studies, AD patients demonstrate a marked impairment in AM compared to their healthy counterparts. This impairment involves a reduced ability to retrieve AMs, a tendency to recall fewer specific details, and longer retrieval times (36, 37, 98). The findings suggest a broad impact of AD on the ability to access and recount personal life experiences, which could potentially serve as an early marker for diagnosis and disease progression monitoring (68).

An important aspect to consider in understanding AM deficits in AD is their relationship with other impaired cognitive domains. AM deficits in AD are strongly linked to impairments in executive functions (36, 98). Research demonstrates that the ability to retrieve and reconstruct specific personal memories depends on executive processes such as organization, planning, and strategic memory search (37, 69). Multiple studies have found significant correlations between executive function measures and AM performance, suggesting that the reduced memory specificity in AD patients stems from executive dysfunction rather than operating in isolation (20, 58). For instance, El Haj et al. (99) observed that cognitive flexibility correlated with and predicted the similarity between past and future thinking in both AD and HC (20). This highlights the role of executive functions in not only retrieving memories but also in constructing future scenarios based on autobiographical information (38). AD patients, who typically have impaired executive functions, show deficits in both remembering past events and imagining future events. Specifically, AD patients generated fewer internal (episodic) details for both past and future events compared to HC, even when controlling for verbal fluency abilities. Therefore, working memory deficits, common in AD, can impair the ability to integrate and organize details of personal memories (38). This could partly explain the tendency of AD patients to produce more generalized and less coherent autobiographical accounts.

Furthermore, attention and processing speed, often compromised in AD, influence the ability to access and retrieve autobiographical information efficiently (23). This can contribute to the longer retrieval times observed in AD patients during AM tasks. Irish and colleagues (23) demonstrated that AD patients exhibit significant difficulties in recalling contextual details across multiple life epochs, which is likely related to these attentional deficits. The researchers observed that source memory deficits due to frontal dysfunction characteristic of the AD pathological process disrupt autonoetic event recall. This frontal impairment is particularly relevant as these regions normally facilitate access to sensory and perceptual details stored in posterior brain regions through the personal knowledge base. Consequently, AD patients develop an impoverished capacity to generate the egocentric or self-referential imagery necessary for rich autobiographical recall, resulting in the production of fragmented and depersonalized accounts of what were once emotionally evocative events. These attentional and processing limitations represent another dimension of cognitive dysfunction that contributes to the overall AM impairment profile in AD, interacting with the memory consolidation and retrieval deficits previously discussed.

Interestingly, Benjamin et al. (107) found that in AD patients, there was a significant positive correlation between semantic fluency and AM performance (107). This suggests that the ability to generate and organize semantic information may support AM retrieval, underscoring the complex interplay between various cognitive processes in AM function. Interestingly, while semantic fluency was predictive of episodic AM, measures of phonemic fluency and working memory showed no significant relationships with episodic AM retrieval (107). This pattern suggests that the complex interplay between cognitive processes in AM function may be particularly dependent on semantic processing abilities, with clinical implications for developing structured external memory aids that follow hierarchical organization to help compensate for semantic fluency deficits in neurodegenerative conditions (107). This integrated perspective on the relationship between AM and other cognitive domains provides a more nuanced understanding of the cognitive deficits in AD. It suggests that AM impairment is not an isolated symptom but part of a broader pattern of cognitive dysfunction. This holistic view can inform both theoretical models of AD and the development of comprehensive, multi-domain cognitive interventions for patients with AD.

Furthermore, the deficit in retrieving specific autobiographical memories has been associated with a diminished capacity for mental time travel - the ability to mentally relive past events (98). When individuals with AD struggle to access detailed AMs, their recollections tend to lose contextual richness and become more abstract or generalized. This process transforms their memory experience from vivid mental recreation of past events to merely recognizing that something happened without the accompanying phenomenological details (98). This connection highlights the intricate relationship between the specificity of autobiographical recall and the phenomenological experience of remembering.

Interestingly, many studies report a temporal gradient in memory impairment among AD patients, often referred to as Ribot’s law (116). This phenomenon describes the relative preservation of remote memories compared to more recent ones, indicating that the disease process may differentially affect memories from different life periods (41, 66). This pattern contrasts with the typical recency effect observed in healthy aging, where recent memories are often more accessible. The findings from Berntsen and colleagues (66) add further complexity to this picture by identifying a “reminiscence bump” in AD patients, showing better preservation of memories from early adulthood (ages 6–30) compared to later periods. This suggests that rather than a simple linear decline in memory access from remote to recent times, AM in AD follows patterns partly reflective of normal memory organization, but without the recency effects typically seen in healthy adults. These memory patterns may be related to how the disease differentially affects brain structures involved in memory consolidation and retrieval, as well as the possible protective role of repeated rehearsal and emotional significance of early life events.

When examining the components of AM, research indicates that personal semantic memory (factual knowledge about one’s life) tends to be better preserved in AD patients compared to episodic memory (recall of specific events) (90). However, it’s important to note that both components show impairment relative to HC, suggesting a widespread effect of AD on AM systems. The study by Seidl and colleagues (90) demonstrates a clear dissociation between semantic and episodic autobiographical memory, with episodic memories—particularly the richness of details—being impaired early in the course of AD or even in the preclinical phase (MCI), while semantic memories remain relatively intact until moderate stages of the disease. This pattern aligns with the multiple trace theory, which proposes separate neural systems for semantic and episodic memories. While semantic memories become gradually independent from hippocampal structures through ‘corticalization,’ episodic memory retrieval requires ongoing reconstructive interaction between the hippocampus and neocortex. The research also identified interesting temporal gradients, with moderate and severe AD patients showing reduced semantic recall for recent periods compared to remote ones, supporting Ribot’s gradient (116).

Neuroimaging studies have provided valuable insights into the neural correlates of AM deficits in AD. Several studies have reported associations between AM impairment and structural or functional changes in brain regions such as the hippocampus, medial prefrontal cortex, and posterior cortical areas (51, 52). Of particular interest is the progressive change in hippocampal volume, which follows a continuum from healthy elderly individuals to those with AD. As noted in recent research, hippocampal volume changes gradually intensify, ranging from healthy elderly individuals exhibiting an intact hippocampal structure to aMCI individuals experiencing smaller hippocampal subfields, and finally, to AD patients presenting severe atrophy in all the hippocampal subfields (54). This pattern of hippocampal atrophy may explain the observed deficits in both episodic and semantic aspects of AM. While episodic memory impairment is a well-established feature of AD, the decline in personal semantic memory is also noteworthy. A possible explanation for these findings is that the initial consolidation of personal semantic facts depends on the hippocampus; therefore, it is also susceptible to early hippocampal damage (68). This suggests that even seemingly preserved aspects of AM, such as personal semantic information, may be vulnerable in the early stages of AD due to hippocampal deterioration. These findings not only enhance our understanding of the neurobiological basis of memory deficits in AD but also suggest potential biomarkers for disease progression and targets for intervention. The gradual nature of hippocampal changes also underscores the potential for early detection and intervention, highlighting the importance of longitudinal studies in tracking AM changes from healthy aging through MCI to AD.

The impact of AM impairment extends beyond cognitive function, significantly influencing the quality of life and well-being of AD patients. Studies have shown that difficulties in recalling personal experiences can affect social interactions, sense of identity, and overall autonomy (45, 112). This highlights the profound role that AM plays in maintaining a sense of self and underscores the importance of addressing these deficits in holistic patient care. Understanding these interrelationships is crucial for developing more targeted and effective interventions. Therapeutic approaches aimed at improving executive functions or working memory could have indirect beneficial effects on AM. Likewise, strategies to support AM retrieval could potentially strengthen other cognitive domains.

### 4.1 Future research directions

Looking towards the future, several promising avenues for research and intervention emerge. The use of sensory cues (such as music, odors, or images) to enhance AM retrieval in AD patients has shown encouraging results (61, 63, 95). Future studies could focus on developing and refining these techniques, potentially incorporating digital technologies to support AM in daily life. Additionally, interventions based on reminiscence therapy or life story work could be further explored as ways to maintain AM function and support quality of life in AD patients.

Building on our understanding of AM processes, recent research enables a comprehensive framework for examining AM deficits in AD. The distinction between construction and elaboration phases, established by Addis and colleagues (117), provides one key dimension for understanding AM impairment. Their groundbreaking study demonstrated that memory construction engages the hippocampus early in retrieval, while elaboration activates a broader autobiographical network. Daviddi and colleagues (118) confirmed through meta-analysis that these phases rely on distinct neural substrates, with construction uniquely engaging the ventromedial prefrontal cortex, right hippocampus, and left angular gyrus, whereas elaboration distinctively activates the right inferior frontal gyrus.

Complementing this temporal framework, Conway and Pleydell-Pearce’s (27) distinction between direct and generative retrieval offers another critical dimension. Direct retrieval allows immediate access to episodic details through specific cues, while generative retrieval requires a more effortful, strategic search process. Daviddi et al. (119) expanded this understanding by demonstrating that direct retrieval primarily engages self-referential regions (anteromedial prefrontal cortex and posterior cingulate cortex), while generative retrieval activates the ventromedial prefrontal cortex, associated with schematic memory and strategic search processes.

These complementary frameworks create a powerful model for investigating AM deficits in AD. Since AD typically begins with medial temporal lobe atrophy before progressively involving frontal regions, patients might experience an earlier impairment in direct retrieval (dependent on hippocampal integrity) and initial construction phases, followed by deterioration of generative retrieval and elaboration capabilities (reliant on prefrontal functions) as the disease progresses. Longitudinal studies integrating these perspectives could provide unprecedented insights into the progression of AM deficits in AD, potentially guiding the development of targeted cognitive interventions that address specific retrieval mechanisms and processing stages most vulnerable at different disease stages.

## 5 Limitations

However, it’s important to address the methodological limitations present in the current body of research. Many studies have relatively small sample sizes, which can limit the generalizability of findings.

A limitation of this systematic review is the considerable methodological heterogeneity among the included studies, which substantially hinders direct comparison of results (23). The assessment of AM in AD patients was conducted using a wide array of instruments and approaches. These instruments, ranged from standardized tests such as the AMI (21, 36, 39, 41, 42, 52, 56, 60, 66–76) and the TEMPau Scale, employed in several studies (20, 22, 58, 59, 61–64, 77–80, 98, 99, 101, 103, 112, 113, 115) to specialized instruments like the Self-Defining Memory Task (SDM) (87, 89, 111) and the AMT. Additionally, some studies employed novel or adapted methods focusing on specific aspects of AM, such as self-related statements in odor conditions or involuntary autobiographical memories (65). Various fewer common tools were also utilized, including the Extended AM Interview (54, 90), Autobiographical Fluency Test (56), and the Remember/Know paradigm (53, 78, 91, 92, 120–123).

This diversity in assessment methods reflects the complexity of AM as a cognitive function and the different research questions explored. However, it severely compromises the ability to make direct comparisons across studies. The lack of a standardized approach to measuring AM in AD patients undermines the possibility of drawing robust general conclusions.

Considering this methodological heterogeneity, we opted for a systematic review approach rather than a meta-analysis. This decision allows us to qualitatively synthesize findings from diverse studies, providing a comprehensive overview of the current research landscape while acknowledging the challenges in directly comparing results across different methodologies.

Future research should aim for larger, more diverse samples and standardized assessment protocols to enhance the robustness and comparability of findings.

In conclusion, these findings underscore the significant impact of AD on AM and suggest potential areas for intervention. The observed deficits have profound implications for patients’ daily lives, social relationships, and sense of self. As research progresses, a deeper understanding of AM in AD could lead to improved diagnostic tools, more effective interventions, and ultimately, a better quality of life for individuals living with Alzheimer’s disease.

## Data Availability

The original contributions presented in the study are included in the article/Supplementary material, further inquiries can be directed to the corresponding author/s.

## References

[1] World Health Organization. (2019). Dementia: Key facts. Available online at: https://www.who.int/news-room/fact-sheets/detail/dementia (Accessed June 17, 2024).

[2] Alzheimer’s Association. 2023 Alzheimer’s disease facts and figures. Alzheimers Dement. (2023) 19:1598–695. doi: 10.1002/alz.13016, PMID: 36918389

[3] Gordon BA Blazey TM Su Y Hari-Raj A Dincer A Flores S . Spatial patterns of neuroimaging biomarker change in individuals from families with autosomal dominant Alzheimer’s disease: a longitudinal study. Lancet Neurol. (2018) 17:241–50. doi: 10.1016/S1474-4422(18)30028-0, PMID: 29397305 PMC5816717

[4] Jack CR Lowe VJ Weigand SD . Serial PIB and MRI in normal, mild cognitive impairment and Alzheimers disease: implications for sequence of pathological events in Alzheimers disease. Brain. (2009) 132:1355–65. doi: 10.1093/brain/awp062, PMID: 19339253 PMC2677798

[5] Ito K Ahadieh S Corrigan B French J Fullerton T Tensfeldt T. Disease progression meta-analysis model in Alzheimer’s disease. Alzheimer’s Dementia. (2010) 6:39–53. doi: 10.1016/j.jalz.2009.05.665, PMID: 19592311

[6] Jack CR Knopman DS Jagust WJ . Tracking pathophysiological processes in Alzheimer’s disease: an updated hypothetical model of dynamic biomarkers. Lancet Neurol. (2013) 12:207–16. doi: 10.1016/S1474-4422(12)70291-0, PMID: 23332364 PMC3622225

[7] Qian J Hyman BT Betensky RA. Neurofibrillary tangle stage and the rate of progression of Alzheimer symptoms: modeling using an autopsy cohort and application to clinical trial design. JAMA Neurol. (2017) 74:540–8. doi: 10.1001/jamaneurol.2016.5953, PMID: 28288263 PMC5547572

[8] Grilli MD Wank AA Bercel JJ Ryan L. Evidence for reduced autobiographical memory episodic specificity in cognitively Normal middle-aged and older individuals at increased risk for Alzheimer’s disease dementia. J Int Neuropsychol Soc. (2018) 24:1073–83. doi: 10.1017/S1355617718000577, PMID: 30136918 PMC6237636

[9] Boyraz FU Er N. Alzheimer ve depresyon tanili gruplar ile normal örneklemde, kisisel ve toplumsal olaylara iliskin otobiyografik bellek özellikleri. Türk Psikoloji Dergisi. (2007) *22*:45.

[10] Spaan PEJ Raaijmakers JGW Jonker C. Alzheimer’s disease versus normal ageing: a review of the efficiency of clinical and experimental memory measures. J Clin Exp Neuropsychol. (2003) 25:216–33. doi: 10.1076/jcen.25.2.216.13638, PMID: 12754679

[11] El Haj M Antoine P Nandrino JL Kapogiannis D. Autobiographical memory decline in Alzheimer’s disease, a theoretical and clinical overview. Ageing Res Rev. (2015) 23:183–92. doi: 10.1016/j.arr.2015.07.001, PMID: 26169474 PMC5456120

[12] Frisone F Brizzi G Sansoni M di Natale AF Pizzoli SFM Stanghellini G . Autobiographical memory in feeding and eating disorders: a systematic review. Psychopathology. (2024) 58:1–25. doi: 10.1159/000540901, PMID: 39378858

[13] Tulving E. Episodic memory: from mind to brain. Annu Rev Psychol. (2002) 53:1–25. doi: 10.1146/annurev.psych.53.100901.135114, PMID: 11752477

[14] Conway MA. Sensory-perceptual episodic memory and its context: autobiographical memory. Phil Trans Royal Soc B. (2001) 356:1375–84. doi: 10.1098/rstb.2001.0940, PMID: 11571029 PMC1088521

[15] Rubin DC. A basic-systems approach to autobiographical memory. Curr Dir Psychol Sci. (2005) 14:79–83.

[16] Berntsen D. Involuntary memories of emotional events: do memories of traumas and extremely happy events differ? Appl Cogn Psychol. (2001) 15:S135–S158. doi: 10.1002/acp.838, PMID: 40304216

[17] Talarico JM LaBar KS Rubin DC. Emotional intensity predicts autobiographical mem- ory experience. Mem Cogn. (2004) 32:1118–32. doi: 10.3758/BF03196886, PMID: 15813494

[18] Fitzgerald JM Broadbridge CL. Latent construct of the autobiographical memory questionnaire: a recollection-belief model of autobiographical experience. Memory. (2013) 21:230–48. doi: 10.1080/09658211.2012.725736, PMID: 23013492

[19] Conway MA Singer JA Tagini A. The self and autobiographical memory: correspondence and coherence. Soc Cogn. (2004) 22:491–529. doi: 10.1521/soco.22.5.491.50768

[20] El Haj M Antoine P Kapogiannis D. Flexibility decline contributes to similarity of past and future thinking in Alzheimer’s disease. Hippocampus. (2015) 25:1447–55. doi: 10.1002/hipo.22465, PMID: 25850800 PMC5460916

[21] Barnabe A Whitehead V Pilon R Arsenault-Lapierre G Chertkow H. Autobiographical memory in mild cognitive impairment and Alzheimer’s disease: a comparison between the Levine and Kopelman interview methodologies. Hippocampus. (2012) 22:1809–25. doi: 10.1002/hipo.22015 22488637

[22] El Haj M Fasotti L Allain P. The involuntary nature of music-evoked autobiographical memories in Alzheimer’s disease. Conscious Cogn. (2012) 21:238–46. doi: 10.1016/j.concog.2011.12.005, PMID: 22265372

[23] Irish M Hornberger M Lah S Miller L Pengas G Nestor PJ . Profiles of recent autobiographical memory retrieval in semantic dementia, behavioural-variant frontotemporal dementia, and Alzheimer’s disease. Neuropsychologia. (2011) 49:2694–702. doi: 10.1016/j.neuropsychologia.2011.05.017, PMID: 21658396

[24] Hannesdottir K Morris RG. Primary and secondary anosognosia for memory impairment in patients with Alzheimer’s disease. Cortex. (2007) 43:1020–30. doi: 10.1016/S0010-9452(08)70698-1, PMID: 17941357

[25] Morris RG Mograbi DC. Anosognosia, autobiographical memory and self knowledge in Alzheimer’s disease. Cortex. (2013) 49:1553–65. doi: 10.1016/j.cortex.2012.09.006, PMID: 23117055

[26] Janssen SMJ Rubin D Conway M. The reminiscence bump in the temporal distribution of the best football players of all time: Pelé, Cruijff or Maradona? Q J Exp Psychol. (2012) 65:165–78. doi: 10.1080/17470218.2011.606372, PMID: 21939366

[27] Conway MA Pleydell-Pearce CW. The construction of autobiographical memories in the self-memory system. Psychol Rev. (2000) 107:261–88. doi: 10.1037/0033-295X.107.2.261, PMID: 10789197

[28] Rubin DC Wenzel AE Anderson J . One hundred years of forgetting: a quantitative description of retention. Psychol Rev. (1996) 103:734–60. doi: 10.1037//0033-295X.103.4.734

[29] Pillemer DB. Momentous events and the life story. Rev Gen Psychol. (2001) 5:123–34. doi: 10.1037/1089-2680.5.2.123

[30] Damoiseaux JS Prater KE Miller BL Greicius MD. Functional connectivity tracks clinical deterioration in Alzheimer’s disease. Neurobiol Aging. (2012) 33:828.e19–30. doi: 10.1016/j.neurobiolaging.2011.06.024, PMID: 21840627 PMC3218226

[31] Cabeza R St JP. Functional neuroimaging of autobiographical memory. Trends Cogn Sci. (2007) 11:219–27. doi: 10.1016/j.tics.2007.02.005, PMID: 17382578

[32] Moher D Liberati A Tetzlaff J Altman DG. Preferred reporting items for systematic reviews and Meta-analyses: the PRISMA statement. Annal Int Med. (2009) 151:264–9. doi: 10.7326/0003-4819-151-4-200908180-00135, PMID: 21603045 PMC3090117

[33] Boyacioglu I Akfirat S. Development and psychometric properties of a new measure for memory phenomenology: the autobiographical memory characteristics questionnaire. Memory. (2015) 23:1070–92. doi: 10.1080/09658211.2014.953960, PMID: 25202835

[34] Ouzzani M Hammady H Fedorowicz Z Elmagarmid A. Rayyan-a web and mobile app for systematic reviews. Syst Rev. (2016) 5:210. doi: 10.1186/s13643-016-0384-4, PMID: 27919275 PMC5139140

[35] Yang B Mallett S Takwoingi Y Davenport CF Hyde CJ Whiting PF . QUADAS-C: a tool for assessing risk of Bias in comparative diagnostic accuracy studies. Ann Intern Med. (2021) 174:1592–9. doi: 10.7326/M21-2234, PMID: 34698503

[36] Meléndez JC Pitarque A Delhom I Real E Abella M Satorres E. A longitudinal study of episodic and semantic autobiographical memory in amci and alzheimer’s disease patients. Int J Environ Res Public Health. (2021) 18:6849. doi: 10.3390/ijerph18136849, PMID: 34202299 PMC8297234

[37] Irish M Lawlor BA O’Mara SM Coen RF. Impaired capacity for autonoetic reliving during autobiographical event recall in mild Alzheimer’s disease. Cortex. (2011) 47:236–49. doi: 10.1016/j.cortex.2010.01.002, PMID: 20153463

[38] Addis DR Sacchetti DC Ally BA Budson AE Schacter DL. Episodic simulation of future events is impaired in mild Alzheimer’s disease. Neuropsychologia. (2009) 47:2660–71. doi: 10.1016/j.neuropsychologia.2009.05.018, PMID: 19497331 PMC2734895

[39] Meléndez JC Redondo R Torres M Mayordomo T Sales A. Autobiographical memory for the differential diagnosis of cognitive pathology in aging. Geriatr Gerontol Int. (2016) 16:1220–5. doi: 10.1111/ggi.12611, PMID: 26460189

[40] Irish M Kamminga J Addis DR Crain S Thornton R Hodges JR . ‘Language of the past’ – exploring past tense disruption during autobiographical narration in neurodegenerative disorders. J Neuropsychol. (2016) 10:295–316. doi: 10.1111/jnp.12073, PMID: 26014271

[41] Müller S Saur R Greve B . Similar autobiographical memory impairment in long-term secondary progressive multiple sclerosis and Alzheimer’s disease. Mult Scler J. (2013) 19:225–32. doi: 10.1177/1352458512450352, PMID: 22685064

[42] Meeter M Kollen A Scheltens P. Retrograde amnesia for semantic information in Alzheimer’s disease. J Int Neuropsychol Soc. (2005) 11:40–8. doi: 10.1017/S135561770505006X 15686607

[43] Moses A Culpin V Lowe C McWilliam C. Overgenerality of autobiographical memory in Alzheimer’s disease. Br J Clin Psychol. (2004) 43:377–86. doi: 10.1348/0144665042388964, PMID: 15530208

[44] Irish M Addis DR Hodges JR Piguet O. Considering the role of semantic memory in episodic future thinking: evidence from semantic dementia. Brain. (2012) 135:2178–91. doi: 10.1093/brain/aws119, PMID: 22614246

[45] Martinelli P Anssens A Sperduti M Piolino P. The influence of normal aging and alzheimer’s disease in autobiographical memory highly related to the self. Neuropsychology. (2013) 27:69–78. doi: 10.1037/a0030453, PMID: 23148495

[46] Meléndez JC Escudero J Satorres E Pitarque A. Type of memory and emotional valence in healthy aging, mild cognitive impairment, and Alzheimer’s disease. Psicothema. (2019) 1:60–5. doi: 10.7334/psicothema2018.181, PMID: 30664412

[47] Rasmussen KW Berntsen D. Remembering a life: an examination of open-ended life stories and the reminiscence bump in patients with Alzheimer’s disease. Memory. (2023) 31:457–73. doi: 10.1080/09658211.2023.2169466, PMID: 36752129

[48] Rasmussen KW Berntsen D. Deficient semantic knowledge of the life course-examining the cultural life script in Alzheimer’s disease. Mem Cogn. (2022) 50:1–15. doi: 10.3758/s13421-021-01202-0 PMID: 34191273

[49] Kirk M Berntsen D. A short cut to the past: cueing via concrete objects improves autobiographical memory retrieval in Alzheimer’s disease patients. Neuropsychologia. (2018) 110:113–22. doi: 10.1016/j.neuropsychologia.2017.06.034, PMID: 28676268

[50] Philippi N Botzung A Noblet V Rousseau F Després O Cretin B . Impaired emotional autobiographical memory associated with right amygdalar-hippocampal atrophy in Alzheimer’s disease patients. Front Aging Neurosci. (2015) 7:21. doi: 10.3389/fnagi.2015.00021, PMID: 25852541 PMC4360763

[51] Philippi N Noblet V Botzung A Després O Renard F Sfikas G . MRI-based Volumetry correlates of autobiographical memory in Alzheimer’s disease. PLoS One. (2012) 7:e46200. doi: 10.1371/journal.pone.0046200, PMID: 23071546 PMC3468599

[52] Eustache F Piolino P Giffard B Viader F Sayette VDL Baron JC . In the course of time: a PET study of the cerebral substrates of autobiographical amnesia in Alzheimer’s disease. Brain. (2004) 127:1549–60. doi: 10.1093/brain/awh166, PMID: 15102619

[53] Genon S Bahri MA Collette F Angel L d'Argembeau A Clarys D . Cognitive and neuroimaging evidence of impaired interaction between self and memory in Alzheimer’s disease. Cortex. (2014) 51:11–24. doi: 10.1016/j.cortex.2013.06.009, PMID: 23993283

[54] Hirjak D Wolf RC Remmele B Seidl U Thomann AK Kubera KM . Hippocampal formation alterations differently contribute to autobiographic memory deficits in mild cognitive impairment and Alzheimer’s disease. Hippocampus. (2017) 27:702–15. doi: 10.1002/hipo.22726, PMID: 28281317

[55] Starkstein SE Boller F Garau L. A two-year follow-up study of remote memory in Alzheimer’s disease. J Neuropsychiatry Clin Neurosci. (2005) 17:336–41. doi: 10.1176/jnp.17.3.336, PMID: 16179655

[56] Greene JDW Hodges JR. The fractionation of remote memory evidence from a longitudinal study of dementia of Alzheimer type. Brain. (1996) 119:–142. doi: 10.1093/brain/119.1.129, PMID: 8624676

[57] Baird A Gelding R Brancatisano O Thompson WF. A preliminary exploration of the stability of music- and photo-evoked autobiographical memories in people with Alzheimer’s and behavioral variant frontotemporal dementia. Music Sci. (2020) 3:3. doi: 10.1177/2059204320957273, PMID: 40297624

[58] El Haj M Antoine P. Discrepancy between subjective autobiographical reliving and objective recall: the past as seen by Alzheimer’s disease patients. Conscious Cogn. (2017) 49:110–6. doi: 10.1016/j.concog.2017.01.009, PMID: 28171831

[59] El Haj M Kapogiannis D Antoine P. Phenomenological reliving and visual imagery during autobiographical recall in Alzheimer’s disease. J Alzheimers Dis. (2016) 52:421–31. doi: 10.3233/JAD-151122, PMID: 27003216 PMC5147522

[60] Irish M Cunningham CJ Walsh JB Coakley D Lawlor BA Robertson IH . Investigating the enhancing effect of music on autobiographical memory in mild Alzheimer’s disease. Dement Geriatr Cogn Disord. (2006) 22:108–20. doi: 10.1159/000093487, PMID: 16717466

[61] Glachet O El Haj M. Emotional and phenomenological properties of odor-evoked autobiographical memories in Alzheimer’s disease. Brain Sci. (2019) 9:135. doi: 10.3390/brainsci9060135, PMID: 31185649 PMC6627121

[62] Glachet O Moustafa AA Gallouj K El Haj M. Smell your memories: positive effect of odor exposure on recent and remote autobiographical memories in Alzheimer’s disease. J Clin Exp Neuropsychol. (2019) 41:555–64. doi: 10.1080/13803395.2019.1586840, PMID: 30890017

[63] El Haj M Clément S Fasotti L Allain P. Effects of music on autobiographical verbal narration in Alzheimer’s disease. J Neurolinguistics. (2013) 26:691–700. doi: 10.1016/j.jneuroling.2013.06.001

[64] Glachet O El Haj M. Odor is more effective than a visual cue or a verbal cue for the recovery of autobiographical memories in AD. J Clin Exp Neuropsychol. (2021) 43:129–43. doi: 10.1080/13803395.2021.1882392, PMID: 33685342

[65] Glachet O El Haj M. Smell your self: olfactory stimulation improves self-concept in Alzheimer’s disease. Neuropsychol Rehabil. (2022) 32:464–80. doi: 10.1080/09602011.2020.1831553, PMID: 33078674

[66] Berntsen D Kirk M Kopelman MD. Autobiographical memory loss in Alzheimer’s disease: the role of the reminiscence bump. Cortex. (2022) 150:137–48. doi: 10.1016/j.cortex.2022.02.008, PMID: 35390739

[67] Ivanoiu A Cooper JM Shanks MF Venneri A. Patterns of impairment in autobiographical memory in the degenerative dementias constrain models of memory. Neuropsychologia. (2006) 44:1936–55. doi: 10.1016/j.neuropsychologia.2006.01.030, PMID: 16519908

[68] Leyhe T Müller S Milian M Eschweiler GW Saur R. Impairment of episodic and semantic autobiographical memory in patients with mild cognitive impairment and early Alzheimer’s disease. Neuropsychologia. (2009) 47:2464–9. doi: 10.1016/j.neuropsychologia.2009.04.018, PMID: 19409401

[69] Greene JDW Hodges JR Baddeley AD. Autobiographical memory and executive function in early dementia of Alzheimer type. Neuropsychologia. (1995) 33:1647–70. doi: 10.1016/0028-3932(95)00046-1, PMID: 8745122

[70] Müller S Mychajliw C Reichert C Melcher T Leyhe T. Autobiographical memory performance in Alzheimer’s disease depends on retrieval frequency. J Alzheimers Dis. (2016) 52:1215–25. doi: 10.3233/JAD-151071, PMID: 27104895

[71] De Simone MS Fadda L Perri R Aloisi M Caltagirone C Carlesimo GA. Does retrieval frequency account for the pattern of autobiographical memory loss in early Alzheimer’s disease patients? Neuropsychologia. (2016) 80:194–200. doi: 10.1016/j.neuropsychologia.2015.11.024, PMID: 26656564

[72] Hou CE Miller BL Kramer JH. Patterns of autobiographical memory loss in dementia. Int J Geriatr Psychiatry. (2005) 20:809–15. doi: 10.1002/gps.1361, PMID: 16116575

[73] Rathbone CJ Ellis JA Ahmed S Moulin CJA Ernst A Butler CR. Using memories to support the self in Alzheimer’s disease. Cortex. (2019) 121:332–46. doi: 10.1016/j.cortex.2019.09.007, PMID: 31670028

[74] Addis DR Tippett LJ. Memory of myself: autobiographical memory and identity in Alzheimer’s disease. Memory. (2004) 12:56–74. doi: 10.1080/09658210244000423 15098621

[75] Meulenbroek O Rijpkema M Kessels RPC Olde Rikkert MGM Fernández G. Autobiographical memory retrieval in patients with Alzheimer’s disease. NeuroImage. (2010) 53:331–40. doi: 10.1016/j.neuroimage.2010.05.082, PMID: 20570740

[76] Greene JDW Miles K Hodges JR Greene JDW Hodges JR Miles K. Neuropsychology of memory and SPECT in the diagnosis and staging of dementia of Alzheimer type. 243. Berlin: Springer-Verlag. (1996) 175–190. 10.1007/BF02444012 8750558

[77] El Haj M Boudoukha A Moustafa AA Antoine P Allain P Gallouj K. “La vie en rose”: a positive shift of autobiographical memory in Alzheimer’s disease. Arch Gerontol Geriatr. (2020) 86:103953. doi: 10.1016/j.archger.2019.103953, PMID: 31541859

[78] Rauchs G Piolino P Bertran F de la Sayette V Viader F Eustache F . Retrieval of recent autobiographical memories is associated with slow-wave sleep in early AD. Front Behav Neurosci. (2013) 7:114. doi: 10.3389/fnbeh.2013.00114, PMID: 24065896 PMC3776137

[79] El Haj M Antoine P Nandrino JL Gély-Nargeot MC Raffard S. Self-defining memories during exposure to music in Alzheimer’s disease. Int Psychogeriatr. (2015) 27:1719–30. doi: 10.1017/S1041610215000812, PMID: 26018841

[80] El Haj M Gandolphe MC Gallouj K Kapogiannis D Antoine P. From nose to memory: the involuntary nature of odor-evoked autobiographical memories in Alzheimer’s disease. Chem Senses. (2018) 43:27–34. doi: 10.1093/chemse/bjx064, PMID: 29040475 PMC5863564

[81] El Haj M Boudoukha A Antoine P Moustafa AA Gallouj K Allain P. Memories supporting myself: autobiographical memory supports self-continuity in Alzheimer’s disease. J Alzheimers Dis. (2019) 70:1217–24. doi: 10.3233/JAD-190440 31322576

[82] Ahmed S Irish M Loane C Baker I Husain M Thompson S . Association between precuneus volume and autobiographical memory impairment in posterior cortical atrophy: beyond the visual syndrome. Neuroimage Clin. (2018) 18:822–34. doi: 10.1016/j.nicl.2018.03.008 29876268 PMC5988022

[83] Han KH Zaytseva Y Bao Y Pöppel E Chung SY Kim JW . Impairment of vocal expression of negative emotions in patients with Alzheimer’s disease. Front Aging Neurosci. (2014) 6:101. doi: 10.3389/fnagi.2014.00101, PMID: 24904413 PMC4033621

[84] Ramanan S Foxe D El-Omar H . Evidence for a pervasive autobiographical memory impairment in Logopenic progressive aphasia. Neurobiol Aging. (2021) 108:168–78. doi: 10.1016/j.neurobiolaging.2021.09.004, PMID: 34653892

[85] Irish M Hornberger M El Wahsh S . Grey and white matter correlates of recent and remote autobiographical memory retrieval -insights from the dementias. PLoS One. (2014) 9:e113081. doi: 10.1371/journal.pone.0113081, PMID: 25396740 PMC4232597

[86] Donix M Brons C Jurjanz L Poettrich K Winiecki P Holthoff VA. Overgenerality of autobiographical memory in people with amnestic mild cognitive impairment and early Alzheimer’s disease. Arch Clin Neuropsychol. (2010) 25:22–7. doi: 10.1093/arclin/acp098, PMID: 19955095

[87] Blagov PS Singer JA. Four dimensions of self-defining memories (specificity, meaning, content, and affect) and their relationships to self-restraint, distress, and repressive defensiveness. J Pers. (2004) 72:481–511. 15102036 10.1111/j.0022-3506.2004.00270.x

[88] El Haj M Allain P Boutoleau-Bretonnière C Chapelet G Kapogiannis D Ndobo A. Does sex matter? High semantic autobiographical retrieval in women and men with Alzheimer’s disease. Psychol Rep. (2024). doi: 10.1177/00332941221130223i PMC10040469 36165092

[89] Ben Malek H Philippi N Botzung A Cretin B Berna F Manning L . Memories defining the self in Alzheimer’s disease. Memory. (2019) 27:698–704. doi: 10.1080/09658211.2018.1554080, PMID: 30526307

[90] Seidl U Lueken U Thomann PA Geider J Schröder J. Autobiographical memory deficits in Alzheimer’s disease. J Alzheimers Dis. (2011) 27:567–74. doi: 10.3233/JAD-2011-110014 21841262

[91] Rodrigues GR Oliveira DS Foss MP Takayanagui OM. Cross-cultural adaptation and validation of the episodic autobiographic memory interview for Brazilian Portuguese. Arq Neuropsiquiatr. (2015) 73:676–80. doi: 10.1590/0004-282X20150084, PMID: 26222359

[92] Westmacott R Black SE Freedman M Moscovitch M. The contribution of autobiographical significance to semantic memory: evidence from Alzheimer’s disease, semantic dementia, and amnesia. Neuropsychologia. (2004) 42:25–48. doi: 10.1016/S0028-3932(03)00147-7, PMID: 14615074

[93] Cuddy LL Sikka R Silveira K Bai S Vanstone A. Music-evoked autobiographical memories (MEAMs) in alzheimer disease: evidence for a positivity effect. Cogent Psychol. (2017) 4:1277578. doi: 10.1080/23311908.2016.1277578

[94] Lopis D Le Pape T Manetta C Conty L. Sensory cueing of autobiographical memories in normal aging and alzheimer’s disease: a comparison between visual, auditory, and olfactory information. J Alzheimers Dis. (2021) 80:1169–83. doi: 10.3233/JAD-200841, PMID: 33646149 PMC8150461

[95] Baird A Brancatisano O Gelding R Thompson WF. Characterization of music and photograph evoked autobiographical memories in people with Alzheimer’s disease. J Alzheimers Dis. (2018) 66:693–706. doi: 10.3233/JAD-180627, PMID: 30320586

[96] Fromholt P Larsen P Larsen SF. Effects of late-onset depression and recovery on autobiographical memory. J Gerontol B Psychol Sci Soc Sci. (1995) 50B:P74–81. doi: 10.1093/geronb/50B.2.P74, PMID: 7757835

[97] Fromholt P Mortensen DB Torpdahl P Bender L Larsen P Rubin DC. Life-narrative and word-cued autobiographical memories in centenarians: comparisons with 80-year-old control, depressed, and dementia groups. Memory. (2003) 11:81–8. doi: 10.1080/741938171, PMID: 12653490

[98] El Haj M Boutoleau-Bretonnière C Gallouj K. The past as seen by women and men with Alzheimer disease sex differences in autobiographical memory. Alzheimer Dis Assoc Disord. (2020) 34:170–4. doi: 10.1097/WAD.0000000000000363 31913962

[99] El Haj M Antoine P Kapogiannis D. Similarity between remembering the past and imagining the future in Alzheimer’s disease: implication of episodic memory. Neuropsychologia. (2015) 66:119–25. doi: 10.1016/j.neuropsychologia.2014.11.015, PMID: 25448861 PMC5471357

[100] El Haj M Postal V Le Gall D Allain P. Directed forgetting of autobiographical memory in mild Alzheimer’s disease. Memory. (2011) 19:993–1003. doi: 10.1080/09658211.2011.626428, PMID: 22092105

[101] El Haj M Antoine P. Describe yourself to improve your autobiographical memory: a study in Alzheimer’s disease. Cortex. (2017) 88:165–72. doi: 10.1016/j.cortex.2017.01.004, PMID: 28142025

[102] El Haj M Robin F. The fabricated past: intentionally fabricated autobiographical memories in Alzheimer’s disease. Cogn Neuropsychiatry. (2022) 27:273–88. doi: 10.1080/13546805.2022.2036114, PMID: 35125060

[103] El Haj M Kapogiannis D Antoine P. The picture of the past: pictures to cue autobiographical memory in Alzheimer’s disease. J Clin Exp Neuropsychol. (2020) 42:914–23. doi: 10.1080/13803395.2020.1825636, PMID: 33003989 PMC9988368

[104] Sartori G Snitz BE Sorcinelli L Daum I. Remote memory in advanced Alzheimer’s disease. Arch Clin Neuropsychol. (2004) 19:779–89. doi: 10.1016/j.acn.2003.09.007 15288331

[105] Kazui H Hashimoto M Hirono N Imamura T Tanimukai S Hanihara T . A study of remote memory impairment in Alzheimer’s disease by using the family line test. Dement Geriatr Cogn Disord. (2000) 11:–58. doi: 10.1159/000017214 10629363

[106] Pyo G Ala T Kyrouac GA Verhulst SJ. A validity study of the working Group’s autobiographical memory test for individuals with moderate to severe intellectual disability. Res Dev Disabil. (2011) 32:70–4. doi: 10.1016/j.ridd.2010.08.011, PMID: 20875945

[107] Benjamin MJ Cifelli A Garrard P Caine D Jones FW. The role of working memory and verbal fluency in autobiographical memory in early Alzheimer’s disease and matched controls. Neuropsychologia. (2015) 78:115–21. doi: 10.1016/j.neuropsychologia.2015.10.006, PMID: 26443928

[108] Strikwerda-Brown C Shaw SR Hodges JR Piguet O Irish M. Examining the episodic-semantic interaction during future thinking – a reanalysis of external details. Mem Cogn. (2022). doi: 10.3758/s13421-021-01222-w 34401984

[109] Sadek JR White DA Taylor KI . Retrograde amnesia in dementia: comparison of HIV-associated dementia, Alzheimer’s disease, and Huntington’s disease. Neuropsychology. (2004) 18:692–9. doi: 10.1037/0894-4105.18.4.692, PMID: 15506837

[110] Rasmussen KW Salgado S Daustrand M Berntsen D. Using nostalgia films to stimulate spontaneous autobiographical remembering in Alzheimer’s disease. J Appl Res Mem Cogn. (2021) 10:400–11. doi: 10.1016/j.jarmac.2020.11.001

[111] El Haj M Allain P. Self-defining memories and their contribution to the sense of self in Alzheimer’s disease. Curr Alzheimer Res. (2020) 17:508–16. doi: 10.2174/1567205017666200807184942 32851943

[112] El Haj M Gallouj K Antoine P. Autobiographical recall as a tool to enhance the sense of self in Alzheimer’s disease. Arch Gerontol Geriatr. (2019) 82:28–34. doi: 10.1016/j.archger.2019.01.011, PMID: 30710846

[113] El Haj M Boutoleau-Bretonnière C Allain P. Memory of decisions: relationship between decline of autobiographical memory and temporal discounting in Alzheimer’s disease. J Clin Exp Neuropsychol. (2020) 42:415–24. doi: 10.1080/13803395.2020.1744527, PMID: 32223584

[114] Liechti C Caviezel MP Müller S Reichert CF Calabrese P Linnemann C . Correlation between hippocampal volume and autobiographical memory depending on retrieval frequency in healthy individuals and patients with Alzheimer’s disease. J Alzheimers Dis. (2019) 72:1341–52. doi: 10.3233/JAD-190047, PMID: 31743996

[115] El Haj M Gallouj K. Mental imagery and autobiographical memory in Alzheimers disease. Neuropsychology. (2019) 33:609–16. doi: 10.1037/neu0000521, PMID: 30896237

[116] Ribot T. Les maladies de la mémoire. Paris: Baillière (1881).

[117] Addis DR Wong AT Schacter DL. Remembering the past and imagining the future: common and distinct neural substrates during event construction and elaboration. Neuropsychologia. (2007) 45:1363–77. doi: 10.1016/j.neuropsychologia.2006.10.016, PMID: 17126370 PMC1894691

[118] Daviddi S Pedale T Jacques PLS Schacter DL Santangelo V. Common and distinct correlates of construction and elaboration of episodic-autobiographical memory: an ALE meta-analysis. Cortex. (2023) 163:123–38. doi: 10.1016/j.cortex.2023.03.005, PMID: 37104887 PMC10192150

[119] Daviddi S Yaya G Sperduti M Santangelo V. A systematic review and meta-analysis of the neural correlates of direct vs. generative retrieval of episodic autobiographical memory. Neuropsychol Rev. (2024) 1–21. doi: 10.1007/s11065-024-09653-3, PMID: 39653872

[120] Tulving E. Memory and consciousness. Can Psychol. (1985) 26:1–12. doi: 10.1037/h0080017

[121] Gardiner JM. Functional aspects of Recollective experience. Mem Cogn. (1988) 16:309–13. doi: 10.3758/BF03197041, PMID: 3210971

[122] Knowlton BJ. The relationship between remembering and knowing: a cognitive neuroscience approach. Acta Psychol. (1998) 98:253–65. doi: 10.1016/S0001-6918(97)00045-0, PMID: 9621833

[123] Markowitsch HJ Thiel A Kessler J Stockhausen HM Heiss WD. Ecphorizing semi-conscious information via the right temporopolar cortex: a PET study. Neurocase. (1997) 3:445–9. doi: 10.1002/wcs.1617

