# Supplementary material for: Autobiographical memory in Alzheimer’s disease: a systematic review
Source: Front Neurol. 2025 Jun 16;16:1546984. doi: 10.3389/fneur.2025.1546984 (PMC12207997; doi:10.3389/fneur.2025.1546984)
Supplement: Supplementary file 1 [file Table_1.pdf]

Supplementary Table 1. PRISMA Checklist for Systematic Review of Autobiographical Memory in Alzheimer's Disease

Reference and year	Could the selection of patients have introduced bias?	Could the conduct or interpretation of the index test have introduced bias?	Could the reference standard, its conduct, or its interpretation have introduced bias?	Could the patient flow have introduced bias?	Notes
Meléndez et al., 2021.	Low	Low	Low	High	Significant dropout of participants between baseline and follow-up, especially in aMCI and AD groups. Potential attrition bias.
Berntsen et al., 2022	Low	Low	Low	Unclear	All participants completed the AMI, but it's not clear if there were any delays between diagnosis and testing that could have affected results.
El Haj et al., 2020 la vie en rose	Low	Low	Low	Low	
Baird et al., 2018	Low	Low	Low	Low	
Seidl et al., 2011	Low	Low	Low	Low	
El Haj et al., 2022	Low	Low	Low	Low	
Greene et al., 1995	Low	Low	Low	Low	
Müller et al., 2013	Low	Low	Low	Low	
Ben Malek et al., 2019	Low	Low	Low	Low	
El Haj et al., 2015	Low	Unclear	Low	Low	
Irish et al., 2012	Low	Low	Low	Low	
El Haj et al., 2019 Autobiographical recall	Low	Low	Low	Low	
Eustache et al., 2004	Low	Low	Unclear	Low	The autobiographical memory test was described, but the reliability and validity of this novel task were not fully established. However, it was based on previous validated measures.
Irish et al., 2016	Low	Low	Low	Low	
Baird et al, 2020	Low	Low	Low	Low	

Supplementary Table 1. PRISMA Checklist for Systematic Review of Autobiographical Memory in Alzheimer's Disease					
**Reference and year**	**Could the selection of patients have introduced bias?**	**Could the conduct or interpretation of the index test have introduced bias?**	**Could the reference standard, its conduct, or its interpretation have introduced bias?**	**Could the patient flow have introduced bias?**	**Notes**
**Kirk et al., 2018**	Low	Unclear	Low	Low	The intervention (Immersive Reminiscence Therapy) was well-described, but due to its nature, it was not possible to blind participants or facilitators to group allocation.
**Starkstein et al., 2005**	Low	Low	Low	High	Significant dropout of participants
**Pyo et al., 2011**	Low	Low	Low	Unclear	Possible effect of drop-out in follow-up
**El Haj et al., 2020 The past**	Low	Low	Low	Low	
**Ahmed et al.,2018**	Low	Low	Low	Low	
**Strikwerda-Brown et al., 2022**	Low	Low	Low	Low	
**Meléndez et al., 2016**	Low	Low	Unclear	Low	While diagnostic criteria for aMCI and AD are mentioned, the exact process of how the final diagnoses were made is not fully detailed. It's unclear if the reference standard results were interpreted without knowledge of the AMI results.
**Barnabe, A. et al., 2012**	Low	Low	Low	Low	
**Müller, S. et al., 2016**	Low	Low	Low	Low	
**Meulenbroek, O. et al., 2010**	Low	Low	Low	Low	
**Genon, S. et al., 2014**	Low	Low	Low	Low	
**Liechti, C. et al., 2019**	Low	Low	Low	Low	
**Rodrigues, G. R. et al., 2015**	Low	Low	Low	Low	
**Rasmussen, K. W., and Berntsen, D., 2022**	Low	Low	Low	Low	
**El Haj et al., 2017**	Unclear	Low	Low	Low	unclear selection of controls
**El Haj, 2011**	Unclear	Low	Low	Low	unclear selection of controls

Supplementary Table 1. PRISMA Checklist for Systematic Review of Autobiographical Memory in Alzheimer's Disease												
**Reference and year**	**Could the selection of patients have introduced bias?**	**Could the conduct or interpretation of the index test have introduced bias?**	**Could the reference standard, its conduct, or its interpretation have introduced bias?**	**Could the patient flow have introduced bias?**	**Notes**							
**El Haj et al., 2017 Descrepancy**	Unclear	Low	Low	Low	unclear selection of controls							
**De Simone et al., 2016**	Low	Low	Low	Low								
**Fromholt, P. et al., (1995).**	High	High	Unclear	Unclear	The selection criteria for "non-demented" centenarians were not well-defined, and the comparison groups were from earlier investigations.	The life narrative method and scoring of memories involve subjective interpretation, and the interviewers were not blinded to participant groups.	The study doesn't explicitly mention a reference standard for diagnosing dementia or depression in the comparison groups.	The study doesn't provide clear information about dropouts or exclusions, and there's a lack of clarity about the time gap between data collection for different groups.				
**El Haj et al., 2013**	Unclear	Low	Low	Low	They retained only participants with recorded autobiographical production,							
**Glachet et al., 2019**	Low	Low	Low	Low								
**Addis, D. R. et al., 2009**	Low	Low	Low	High	Different setting test conduction							
**Ramanan et al., 2021**	Low	Low	Low	Low								
**El Haj et al., 2015 Flexibility**	Unclear	Low	Unclear	Low	unclear selection of controls and of the diagnostic process							

Supplementary Table 1. PRISMA Checklist for Systematic Review of Autobiographical Memory in Alzheimer's Disease

Reference and year	Could the selection of patients have introduced bias?	Could the conduct or interpretation of the index test have introduced bias?	Could the reference standard, its conduct, or its interpretation have introduced bias?	Could the patient flow have introduced bias?	Notes
**El Haj et al., 2018**	Low	Unclear	Low	Low	The volume level for music was adjusted during each session, which could introduce some variability. The study doesn't mention if the researchers conducting the tests were blinded to the participants' diagnoses. There's no mention of whether the order of odor presentation (coffee vs. vanilla) was randomized.
**Irish et al., 2014**	Low	Low	Low	Low	
**Hirjak et al., 2017**	Low	Low	Low	Low	
**Irish et al., 2011 impaired capacity**	Low	Unclear	Low	Low	The length of the test session varied between groups (60 min for middle-aged controls, 90-120 min for elderly controls, 60-90 min for AD patients), which could potentially introduce some bias.
**Philippi et al., 2015**	Low	Low	Low	Low	
**Leyhe et al., 2009**	High	Low	Unclear	Low	Unclear patient recruitment, no mention of random sampling, MMSE scores differences

Supplementary Table 1. PRISMA Checklist for Systematic Review of Autobiographical Memory in Alzheimer's Disease												
**Reference and year**	**Could the selection of patients have introduced bias?**	**Could the conduct or interpretation of the index test have introduced bias?**	**Could the reference standard, its conduct, or its interpretation have introduced bias?**	**Could the patient flow have introduced bias?**	**Notes**							
**Han et al., 2014**	High	Unclear	Low	Unclear	The study included 21 AD patients and 19 healthy controls, but the selection process is not clearly described. Exclusion criteria are mentioned, but it's unclear how participants were recruited or if it was consecutive or random.The small sample size increases the risk of selection bias.	The index test is the evaluation of emotional expression in autobiographica l memory retrieval. While the procedure is described, it's not clear if the evaluators were blinded to the clinical information of the participants. <The subjectivity of the emotional expression rating (0-2 scale) may introduce bias.	The study doesn't clearly state the time interval between the reference standard (AD diagnosis) and the index test (emotional expression evaluation).It's not mentioned if all participants received the same reference standard tests. <No information is provided about any participants who might have dropped out or been excluded from the analysis.					
**Irish et al., 2006**	High	Low	Unclear	Low	Small sample size, It's unclear if the selection was consecutive or random, the exclusion criteria may have introduce bias	it's not clear if the reference standard results were interpreted without knowledge of the index test results.						
**Fromholt et al., 2003**	Unclear	Unclear	Unclear	High	unclear selection of controls	it's not clear if the evaluators were blinded to the clinical information of the participants.	it's not clear if the evaluators were blinded to the clinical information of the participants.	Different setting test conduction				

Supplementary Table 1. PRISMA Checklist for Systematic Review of Autobiographical Memory in Alzheimer's Disease												
**Reference and year**	**Could the selection of patients have introduced bias?**	**Could the conduct or interpretation of the index test have introduced bias?**	**Could the reference standard, its conduct, or its interpretation have introduced bias?**	**Could the patient flow have introduced bias?**	**Notes**							
**El Haj et al., 2019 Memories Supporting Myself:**	Unclear	Unclear	Unclear	Low	unclear selection of controls	The TALE questionnaire was used as the primary measure. While it's a validated tool, it's unclear if the administrators were blinded to participant group. The translation process is described, but potential impacts are not discussed.	the study doesn't provide details on how the criteria were applied or if there was any blinding of the clinicians to other test results.					
**El Haj et al., 2020 memory of decisions**	Low	Low	Low	Unclear	No deteiled information regarding flow							
**Rose Addis et al., 2004**	High	Unclear	Low	Low	Only memory clinic patients, exclusion criteria may have introduced bias, No clear information regarding control group	The study used multiple tests for autobiographica l memory and identity, but it's not clear if these were administered in a standardized way.	Some tests were modified for use with AD patients (e.g., Twenty Statements Test), which could introduce bias.	It's unclear if the assessors were blinded to the participants' group status.				
**El Haj et al., 2019 Mental imagery**	High	Low	Unclear	Low	unclear selection of participants and controls	It's unclear if the reference standard was applied independently of the index test results.						
**Philippi et al., 2011**	High	Low	Unclear	Low	Eterogeneity in AD disease, no randomization, unclear selection of participants		However, it's not clear if the diagnosis was made independently of the autobiographica l memory assessment.	The article doesn't specify if the clinicians making the diagnosis were blinded to the autobiographica l memory test results.				

Supplementary Table 1. PRISMA Checklist for Systematic Review of Autobiographical Memory in Alzheimer's Disease												
**Reference and year**	**Could the selection of patients have introduced bias?**	**Could the conduct or interpretation of the index test have introduced bias?**	**Could the reference standard, its conduct, or its interpretation have introduced bias?**	**Could the patient flow have introduced bias?**	**Notes**							
**Cuddy et al., 2017**	High	Unclear	Unclear	High	Eterogeneity in participants, Some AD participants were excluded as "nonresponders," which could introduce bias.	The "index test" in this case would be the music excerpts used to evoke autobiographical memories which is subjective	Two independent judges categorized the memories, which helps reduce bias, but agreement was only moderate (Cohen's κ = 0.59).	Some AD participants (4 out of 24) were unable to provide any memories and were excluded from further analysis.	Not all AD participants completed all supplementary tests, which could introduce bias in those analyses.			
**Greene et al.,1995** Neuropsychology of memory	Low	Unclear	Unclear	Low	The researchers were not explicitly blinded to the clinical status of participants when interpreting SPECT results, which could potentially introduce bias.	The authors acknowledge that some patients initially included as DAT were later excluded when they showed no progression, indicating potential misclassification.						
**Glachet et al, 2021**	Low	Low	Unclear	Low	While the study compared odor-evoked memories to those evoked by visual and verbal cues, there's no clear "gold standard" for autobiographical memory assessment. The comparison to other cue types provides useful information, but it's not a definitive reference standard.							

Supplementary Table 1. PRISMA Checklist for Systematic Review of Autobiographical Memory in Alzheimer's Disease													
**Reference and year**	**Could the selection of patients have introduced bias?**	**Could the conduct or interpretation of the index test have introduced bias?**	**Could the reference standard, its conduct, or its interpretation have introduced bias?**	**Could the patient flow have introduced bias?**	**Notes**								
**Moses et al., 2004**	High	Unclear	Unclear	Unclear	Reduced sample, no blinding process, unclear control selection, no randomization	However, the researchers were not blinded to the participants' group (AD or control), which could potentially influence their interpretation.	However, the study doesn't mention if the diagnosis was confirmed by multiple clinicians or if there was any blinding in the diagnostic process.	t's not clear if there was any time gap between the AD diagnosis and the study procedures, which could potentially affect results if cognitive decline progressed.					
**Donix et al., 2010**	Low	Low	Unclear	Low	Unclear control selection								
**Hou et al., 2005**	Low	Low	Low	Low									
**Ivanoiu et al., 2006**	High	Unclear	High	High	The study includes a small sample size, especially for the SD patients (only 2 cases). The selection criteria for AD patients and controls are not clearly specified. There's a significant age difference between the AD group and the control group, which could introduce bias. The education level also differs significantly between groups.	The scoring method, while described in detail, involves some subjective elements, especially in scoring episodic memories. The scoring method, while described in detail, involves some subjective elements, especially in scoring episodic memories.	There is no clear reference standard for autobiographical memory assessment. The diagnosis of AD and SD is based on clinical criteria, but the exact methods of diagnosis are not fully described. The lack of a definitive biomarker or pathological confirmation of diagnoses increases the risk of misclassification.	The study doesn't clearly state if all patients underwent the same assessment procedures in the same order. The time between clinical diagnosis and ABM testing is not specified. For the moderate SD patient, testing was carried out in two sessions with a 2-week gap, which differs from the other participants and could introduce variability.				.
**El Haj et al., 2016**	Low	Unclear	Not applicable	Low	Subjective interpretation								
**Irish et al., 2011 profiles**	Low	Low	Low	Low									

Supplementary Table 1. PRISMA Checklist for Systematic Review of Autobiographical Memory in Alzheimer's Disease												
**Reference and year**	**Could the selection of patients have introduced bias?**	**Could the conduct or interpretation of the index test have introduced bias?**	**Could the reference standard, its conduct, or its interpretation have introduced bias?**	**Could the patient flow have introduced bias?**	**Notes**							
**Rasmussen et al., 2023**	High	Unclear	Unclear	High	The study included 21 individuals with Alzheimer's disease (AD) and 22 healthy controls. Patients were recruited from a hospital-based specialized memory clinic, which may not represent the full spectrum of AD patients. The control group was age-matched, but it's unclear how they were recruited or if they are representative of the general population. Exclusion criteria were mentioned (e.g., vision/hearing disability, substance abuse, psychiatric/neurological illness), but it's not clear how these were assessed or if they were applied equally to both groups.	the open-ended nature of the task might introduce variability in how participants approached it.	However, it's not clear if the diagnoses were confirmed by multiple clinicians or if there was any follow-up to confirm the diagnosis.	The study mentions that AD participants were tested in two separate sessions to avoid fatigue, while controls completed all tasks in one session. This difference in testing conditions could potentially introduce bias.				
**Sartori et al., 2004**	High	Low	Unclear	Low	The study selected only 10 DAT patients from a larger sample of 80, based on specific MMSE scores and absence of other illnesses. This selective process might not represent the full spectrum of DAT patients.		The study doesn't provide details on how the healthy controls were confirmed to be free of cognitive impairment beyond MMSE scores.					

Supplementary Table 1. PRISMA Checklist for Systematic Review of Autobiographical Memory in Alzheimer's Disease

Reference and year	Could the selection of patients have introduced bias?	Could the conduct or interpretation of the index test have introduced bias?	Could the reference standard, its conduct, or its interpretation have introduced bias?	Could the patient flow have introduced bias?	Notes	
Kazui et al., 2000	Low	Unclear	Low	Low	While the FLT is well-described, it's not clear if there was blinding during interpretation. The lack of a pre-specified threshold for a positive result could introduce bias.	
Rauchs et al., 2013	Low	Low	Low	Low		
Meeter et al., 2005	High	Low	Low	Low	Control study, small sample size, strict exclusion criteria	
Sadek et al., 2004	High	Low	Unclear	Low	There are significant age differences between the groups (HIV-D and HD patients were younger than AD patients).	There isn't a true "reference standard" in this study as it's not a diagnostic accuracy study.

Supplementary Table 1. PRISMA Checklist for Systematic Review of Autobiographical Memory in Alzheimer's Disease

Reference and year	Could the selection of patients have introduced bias?	Could the conduct or interpretation of the index test have introduced bias?	Could the reference standard, its conduct, or its interpretation have introduced bias?	Could the patient flow have introduced bias?	Notes							
**Haj et al., 2020**	High	Unclear	Low	Low	The study included 30 patients with probable Alzheimer's disease and 30 healthy older adults, but the selection process is not clearly described. There is no mention of consecutive or random sampling. Exclusion criteria are listed, but it's unclear how many potential participants were excluded based on these criteria. The controls were often spouses, relatives, or friends of the AD participants, which could introduce selection bias.	The self-defining memory task is described, but there is limited information on standardization of administration. While coding instructions are mentioned, it's unclear if the raters were blinded to participant group. While coding instructions are mentioned, it's unclear if the raters were blinded to participant group.						

Supplementary Table 1. PRISMA Checklist for Systematic Review of Autobiographical Memory in Alzheimer's Disease												
**Reference and year**	**Could the selection of patients have introduced bias?**	**Could the conduct or interpretation of the index test have introduced bias?**	**Could the reference standard, its conduct, or its interpretation have introduced bias?**	**Could the patient flow have introduced bias?**	**Notes**							
**El Haj et al., 2015**	High	Unclear	Unclear	Unclear	The exclusion criteria (e.g., psychiatric illness, clinical depression) may have eliminated participants with comorbidities common in AD populations. Participants were recruited from local retirement homes and the community, which could introduce selection bias. Several AD patients were excluded due to health problems, personal reasons, auditory deficiencies, or aphasic deficiencies, potentially skewing the sample.	The addition of a 5-point category for self-defining memories to the TEMPau scale was not validated.	However, the study doesn't mention if the assessors of the reference standard (AD diagnosis) were blinded to the results of the autobiographica l memory tests.	The study had three sessions separated by approximately one week, which could introduce variability due to day-to-day changes in participants' conditions. There was a high dropout rate among AD participants (14 out of 36 originally recruited), which could affect the results.				
**Lopis et al., 2021**	Low	Unclear	Low	Low	the study does not explicitly mention blinding of the experimenters to participant group, which could potentially introduce some bias.							
**Glachet et al., 2019**	Low	Unclear	Low	Low	it's not clear if the main rater was blinded to participant group, which could introduce some bias.							

Supplementary Table 1. PRISMA Checklist for Systematic Review of Autobiographical Memory in Alzheimer's Disease

Reference and year	Could the selection of patients have introduced bias?	Could the conduct or interpretation of the index test have introduced bias?	Could the reference standard, its conduct, or its interpretation have introduced bias?	Could the patient flow have introduced bias?	Notes							
**Glachet et al., 2022.**	Low	Unclear	Low	Low	it's unclear if the researchers coding the responses were blinded to participant group.							
**Westmacott et al, 2004**	High	Unclear	High	Low	potential for selection bias due to the small, heterogeneous sample that may not be representative.	no mention of blinding of test administrators or interpreters.	The classification of "High R" vs "Low R" names was based on ratings from control participants, not an objective standard. This subjective classification method could introduce bias.					
**El Haj et al., 2022 the fabricated past**	High	Unclear		Unclear	AD patients were recruited from retirement homes while controls were from community centers, which could introduce differences beyond AD status. Some AD patients were excluded because they couldn't construct fabricated memories, potentially biasing the sample towards higher-functioning AD patients.	The assessment of vividness relies on self-report, which could be influenced by the participants' understanding and interpretation of the scale.	However, the study doesn't provide details on how recently the diagnoses were made or if they were confirmed for the study. It's not clear if the researchers conducting the memory tasks were blinded to the diagnostic status of the participants.	It's not clear if all participants completed all parts of the study or if there were any dropouts. The time between AD diagnosis and study participation is not reported, which could be relevant if cognitive status changed significantly. The time between AD diagnosis and study participation is not reported, which could be relevant if cognitive status changed significantly.				

Supplementary Table 1. PRISMA Checklist for Systematic Review of Autobiographical Memory in Alzheimer's Disease

Reference and year	Could the selection of patients have introduced bias?	Could the conduct or interpretation of the index test have introduced bias?	Could the reference standard, its conduct, or its interpretation have introduced bias?	Could the patient flow have introduced bias?	Notes							
**Greene et al., 1996**	Low	Low	Low	Unclear	the loss of 9 patients (27% of the original DAT group) does raise the risk of bias slightly, hence the low to unclear rating.							
**Martinelli et al., 2013**	Low	Unclear	Low	Low		The reference standard is not explicitly discussed in traditional diagnostic terms, making it unclear.						
**El Haj et al., 2012**	High	Low	Unclear	Low	There's no mention of random selection or consecutive enrollment. The study included three groups: young adults, older adults, and Alzheimer's disease (AD) patients. However, the selection criteria for these groups are not clearly described.	The criteria used to define involuntary memories (specificity, emotional content, retrieval time) are based on previous research, but their validity as a reference standard is not fully established.The study compares memories evoked in silence vs. after music exposure, but it's not entirely clear if this effectively distinguishes between voluntary and involuntary memories.						
**El Haj et al., 2020 The picture of the past**	Low	Low	Low	Low								

Supplementary Table 1. PRISMA Checklist for Systematic Review of Autobiographical Memory in Alzheimer's Disease												
**Reference and year**	**Could the selection of patients have introduced bias?**	**Could the conduct or interpretation of the index test have introduced bias?**	**Could the reference standard, its conduct, or its interpretation have introduced bias?**	**Could the patient flow have introduced bias?**	**Notes**							
**Benjamin et al., 2015**	Low	Unclear	Low	High	However, coding of the VAF 4 task was carried out by the lead investigator who was not blinded to group status, which could introduce bias.	The authors mention that if participants became fatigued, the battery was shortened for ethical reasons. This could potentially introduce some inconsistency in the data collected.						
**Meléndez et al., 2019**	Low	Unclear	Low	Low	it's not explicitly stated whether the judges were blinded to the participants' clinical status, which could potentially introduce bias.							
**Rathbone et al., 2019**	Low	Low	Low	Low								
**Rasmussen et al., 2021**	High	High	Unclear	Low	AD patients were recruited with assistance from regional dementia coordinators and local dementia care workers, which may introduce selection bias. Healthy controls were recruited through local advertising and the research center's participant database, potentially leading to a non-representative sample.	However, the reliance on spontaneous utterances means that some involuntary autobiographical memories may have been experienced but not verbalized, potentially underestimating their occurrence. The experimenters were not blinded to the participants' group status (AD or control), which could introduce bias in their interactions and interpretations.	There is no clear reference standard in this study as it's not a typical diagnostic accuracy study.						
