# Supplementary material for: Autobiographical memory in Alzheimer’s disease: a systematic review
Source: Front Neurol. 2025 Jun 16;16:1546984. doi: 10.3389/fneur.2025.1546984 (PMC12207997; doi:10.3389/fneur.2025.1546984)
Supplement: Supplementary file 2 [file Table_2.pdf]

Supplementary Table 2. The table shows the main results studying AM (AM) in patients with Alzheimers's Disease.

Reference and year	Purpose	Sample Features	Measures for testing autobiographical memory (AM)	Autobiographical memory task	Main outcomes
**Addis et al., 2004**	To investigate the status of AM and identity in individuals with AD and the association between memory impairments and changes with identity.	20 patients with probable AD<br>Mean age: 75.45 (SD 7.06)<br>Sex: F and M<br><br>20 control subjects<br>Mean age: 75.20 (SD 6.81)<br>Sex: F and M	(AMI [Kopelman et al., 1989;1999]<br><br>Autobiographical Fluency task [Dritschel et al., 1992]	AMI: Recalling personal semantic and personal incident memory across three lifetime periods<br><br>Autobiographical Fluency: Generating names of people known and personal events for three lifetime	AD performed significantly worse than HC on both AMI personal semantic ($p < .001$) and personal incident ($p < .001$) components. AD showed temporal gradient for personal semantic memory on AMI ($p = .009$).<br>AD group performed significantly worse on autobiographical fluency for names ($p < .001$) and events ($p < .001$)<br>AD group showed changes in strength ($p < .001$), quality ($p = .011$), and direction ($p < .001$) of identity compared to HC.<br>Impairments in childhood and early adulthood autobiographical memories were associated with changes in strength and quality of identity (multiple correlations significant at $p < .05$)

				periods	
**Addis, D. R. et al., 2009**	To examine the ability of AD patients to generate past and future autobiographical events compared to HC.	16 patients with AD<br>Mean age: 77.06 (SD 8.10)<br>Sex: F and M<br><br>16 control subjects<br>Mean age: 78.75 (SD 5.17)<br>Sex: F and M	Adapted version of AI [Levine et al., 2002]	Past-Future AI Task: Generating 5 past and 5 future events from the past/next few months<br><br>Remote Memory AI Task: Generating events from 5 lifetime periods (early childhood, teenage years, early adulthood, middle adulthood and recent adulthood)	AD generated significantly fewer internal details for both past and future events compared to HC ($p < .001$).<br>No significant difference between groups in external details when controlling for fluency abilities.<br>Initially, AD patients showed fewer details for remote events across all lifetime periods compared to controls ($p < .01$) but this group difference was no longer significant after controlling for fluency abilities and gender.<br>Deficits in AD were evident for both recent and remote autobiographical memories.
**Ahmed et al.,2018**	To investigate the characteristics and	18 patients with typical AD (tAD)<br>Mean age:	AI [Levine et al., 2002]	Recalling and describing specific autobiographi	Both tAD and PCA showed significantly reduced internal (episodic) details compared to HC in both free (tAD p=.001; PCA p=.001) and probed conditions (tAD p=.000; PCA p=.000).<br>tAD patients showed significantly more external details compared to HC in the free

	neuroanatomical substrates of autobiographical memory in patients with posterior cortical atrophy (PCA).	66.9 (SD 8.7)<br>Sex: F and M<br><br>14 patients with PCA<br>Mean age: 64.9 (SD 7.7)<br>Sex: F and M<br><br>28 control subjects<br>Mean age 69.5 (SD 5.7)<br>Sex: F and M		cal events spanning four distinct life periods: Teenage Years, Early Adulthood, Middle Adulthood, and Recent Time.	condition (p=.001) only, whereas PCA showed the same pattern in both free (p=.003) and probed (p=.004) conditions.<br>PCA had a significantly lower internal-to-total detail ratio compared to HC (p=.000) and AD (p=.000).<br>PCA showed specific reductions in spatiotemporal (p=.000) and perceptual (p=.000) details compared to HC.<br>Perceptual detail retrieval in PCA correlated with gray matter density in the right precuneus (p<.05).
**Baird et al, 2020**	To investigate the consistency of Music evoked autobiographical memories (MEAMs) and photo-evoked autobiographical memories (PEAMs) over a six-month period in AD, patients with behavioral variant frontotemporal dementia (Bv-FTD), and healthy controls (HC).	7 patients with probable AD<br>Mean age at T1: 77 (SD14.3)<br>Sex: F and M<br><br>6 patients with Bv-FTD<br>Mean age at T1: 71.7 (SD 10.6)<br>Sex: M<br><br>9 control subjects ,<br>Mean age at T1: 74.8 (SD 9.0)<br>Sex: F and M	MusEQ [Vanstone et al., 2016]	Listening to 16 songs and seeing 16 photos depicting world-famous events across eight decades (1930-2010) evoking and describing memories according to topic content and emotional valence	At T1 the majority of the AD patients evoked more MEAMs than PEAMs, or an equal number of MEAMs than PEAMs while at T2 AD patients and the majority of HC had more or equal number of PEAMs than MEAMs. AD group showed a significant decrease in familiarity ratings for photos evoking memories over time (p = .008).<br>At T1, significant differences were observed in the emotional valence of memories evoked among AD, SD and HC with songs having a more positive emotional tone compared to photographs.

**Baird et al., 2018**	To explore and compare the frequency and specificity of AM evoked by famous songs (MEAMs) versus photographs of famous events (PEAMs) in patients with AD and HC	10 patients with probable AD Mean age: 77.7 (SD 12.7) Sex: F and M<br><br>10 control subjects Mean age: 76.0 (SD 9.3) Sex: F and M	Music Experience Questionnaire (MusEQ) [Vanstone et al., 2016]	Listening to 16 famous songs and observing 16 photographs of famous events from 1930-2010 recalling any memories which were coded for specificity and topic content	AD group showed significantly fewer PEAMs than HC($p < 0.05$).<br>MEAMs were typically less specific than PEAMs in both groups.<br>In the AD, MEAMs were<br>more frequently reported in response to songs from<br>when the participants were aged 10–30 years compared with when they were aged 31–50 years<br>($p = 0.006$) or 51 years and over ($p < 0.001$)
**Barnabe, A. et al., 2012**	To examine autobiographical memory in patients with MCI and AD employing two different interview methodologies<br>.	10 patients with probable AD, Mean age: 79.30 (SD 6.04) Sex: F and M<br><br>20 patients with MCI, Mean age: 76.40 (SD 6.87) Sex: F and M<br><br>20 control subjects Mean age:	AMI [Kopelman et al, 1989], AI [Levine et al., 2001]	Recalling autobiographical memories from 5 life periods and answering questions about personal semantic information.	AD were impaired on both episodic and semantic autobiographical memory compared to HC on the AMI ($p < 0.01$).<br>Both AD and MCI showed deficits in internal (episodic) details on the AI compared to HC ($p < 0.001$).<br>MCI showed impaired episodic but intact semantic autobiographical memory on the AMI compared to HC ($p < 0.01$).<br>A temporal gradient in episodic memory was found for AD, with better recall of remote vs. recent memories ($p < 0.05$).<br>The methodology used (AMI vs AI) impacted the detection of temporal gradients and patterns of impairment.

		78.35 (SD 5.75)<br><br>Sex: F and M			
**Ben Malek et al., 2019**	To explore the relationship between self-defining memories (SDMs), integrative meaning, and self-concept in early-stage AD.	15 patients with AD<br>Mean age: 74.06 (SD 5.96)<br>Sex: F and M<br><br>15 control subjects<br>Mean age: 72.73 (SD 4.77)<br>Sex: F and M	Self-defining memory task (SDM) [Blagov & Singer, 2004]	Recalling and describing 5 SDMs that had to be<br>-older than one year<br>-Highly vivid<br>-Associated with intense emotions<br>-Frequently rehearsed<br>-Related to important aspects of their personal life	Individuals with AD generated significantly fewer memories that were spontaneously integrated, compared to HC ($p = 0.05$).<br>AD patients integrated fewer memories than HC ($p = 0.03$) and the integrative meaning was significantly less frequently tied to the self ($p = 0.006$)<br>Patients with AD demonstrated a weaker and less complex sense of self compared to HC, with statistical significance ($p < 0.002$).<br>In AD patients, integrative ability was significantly correlated with complexity of self-concept (, $p < 0.01$).
**Benjamin et al., 2015**	To investigate the relationships between working memory (WM), verbal fluency, and episodic AM retrieval in	10 patients with AD<br>Mean age: 66.2 (SD 11.1)<br>Sex: F and M<br><br>10 Control subjects	Verbal Autobiographical Fluency (VAF) task [Piolino et al.,2010]	The VAF task has 4 stages:<br>VAF1:-Listing general life periods<br>VAF2: Listing general events within a chosen life	AD provided significantly fewer episodic details compared to HC ($p < 0.01$).<br>AD and HC differed significantly on all measures of WM, and semantic fluency, total verbal fluency, but not phonemic fluency.<br>AD scores in VAF3 and in recalling episodic details in VAF4 were significantly lower compared to HC.<br>There was a positive relationship between auditory WM and hierarchical search for life-periods, and between semantic fluency and episodic AM.

	AD patients and HC.	Mean age: 61.4 (SD 11.8)<br>Sex: F and M		period<br>VAF3: Listing specific events within a chosen general event<br>VAF4: Providing details of a specific event	
**Berntsen et al., 2022**	To develop an expanded version of the AMI, which segmented the life-span into seven time-periods (age 0-5, 6-11, 12-19, 20-30, 31-45, 46+, most recent year).	25 patients with probable AD<br>Mean age: 81.12 (SD 6.82)<br>Sex: F and M<br><br>30 control subjects<br>Mean age: 79.87 (SD 6.31)<br>Sex: F and M	AMI expanded version. range from an absence of an answer or purely semantic response to providing a purely episodic event and vividness	Recalling two autobiographical incidents (giving a total of 14 autobiographical incidents for the full interview) from seven periods	There is a significantly elevated recall performance ($p < 0.01$) in the AD between the ages of 6-30, corresponding to a reminiscence bump, when compared to other time periods. After age 30, the performance in the AD group drops dramatically ($p < 0.001$) and remains consistently low ($p > 0.05$ for differences between subsequent time periods) over the remaining lifespan up to the most recent year.
**Cuddy et al., 2017**	To investigate the positive effect of music-evoked autobiographical memories (MEAMs) to enhance recall	20 patients with probable AD<br>Median age: 77.5 years (range 63-89)<br>Sex: F and M	MEAMs assessment:<br>-Word Count Analysis: Using the Linguistic Inquiry Word Count (LIWC) software to	Listening to familiar instrumental music excerpts and elaborating AM from their past.	AD and older control subjects produced similar numbers of MEAMs (no significant difference).<br>AD memories were less specific compared to older control subjects' memories ($p = 0.001$).<br>AD and older control subjects rated their MEAMS as more vivid than younger control subjects ($p < 0.001$)<br>AD and older control subjects rated their MEAMS as more positive ($p < 0.001$) and less negative than younger control subjects ($p = 0.001$).

	in AD.	20 younger control subjects<br>Mean age: 21.2 (SD 1.2)<br>Sex: F and M<br><br>20 older control subjects<br>Mean age: 73.1 (SD 5.2)<br>Sex: F and M	tally positive and negative words in the memory descriptions.<br>-Content Topic Distribution: Categorizing the memories according to both positive and negative topics [Schlagman et al., 2006].<br>-Self-Rating of Valence (positivity and negativity).	If it was evoked, they had to answer follow-up questions about the memory's characteristics (e.g., valence, specificity, vividness, age at the time of the memory).	AD and older control subjects had higher percentage of memories categorized as positive compared to younger control subjects (p = 0.003)
**De Simone et al., 2016**	To assess the impact of memory retrieval frequency on the quality of AM in AD patients, and to evaluate how this relationship	14 patients with AD<br>Mean age: 76.64 (SD 4.24)<br>Sex: F and M<br><br>15 control subjects<br>Mean age: 73.27 (SD	AMI [Kopelman et al., 1989]	Recalling three incidents from each of four time periods (adolescence, early adulthood, middle adulthood,	AD performed significantly worse than HC in recalling both personal episodic and semantic memories (p < .001).<br><br>A temporal gradient was found in AD patients' episodic and semantic memory performances, with better preservation of remote memories than recent ones (p < .001).<br>AD showed a significant effect of retrieval frequency on episodic memory performance (p < .001) which was temporally-graded and inversely related to the recency of memories (p < .01).

	may evolve over the course of the disease.	6.47)<br>Sex: F and M		recent events) and rating the retrieval frequency of each incident. They also answered questions about personal semantic information for each time period.	
**Donix et al., 2010**	To investigate whether people with early AD and aMCI show reduced specificity in AM retrieval compared to HC.	16 patients with AD<br>Mean age: 64.67 (SD 7.3)<br>Sex: F and M<br><br>16 patients with aMCI<br>Mean age: 63.13 (SD 5.78)<br>Sex: F and M<br><br>16 control subjects<br>Mean age: 62.94 (SD 5.73)	ABM task [Williams & Broadbent, 1986]<br><br>Responses were categorized as specific, extended, or categoric.	Being exposed to 10 cue words (5 positive, 5 negative) and retrieving a specific AM for each cue within 60 seconds.	AD produced significantly fewer specific memories than control subjects ($p < .001$).<br>AD produced significantly more categoric ($p < .001$) and extended ($p = .01$) memories than control subjects.<br>aMCI produced significantly fewer specific memories than control subjects($p < .001$) and significantly more extended memories than control subjects ($p = .004$)<br>AD produced significantly more categoric memories and a lower number of specific memories compared to aMCI ($p < .001$).

		Sex: F and M			
**El Haj & Allain, 2020**	To investigate the characteristics of SDMs in patients AD, including their specificity, emotional valence, integration, centrality, and contribution to self-continuity.	30 patients with probable AD=30<br>Mean age: 73.27 (SD 6.04)<br>Sex: F and M<br><br>30 control subjects<br>Mean age: 70.33 (7.77)<br>Sex: F and M	Adaptation of Singer et al.'s classification system for self-defining memories [Singer et al., 1992, 2002, 2004]<br><br>Questions on centrality and self-continuity [Holm et al. 2016]	Recalling two self-defining memories and rating their centrality and contribution to self-continuity.	AD produced fewer specific and more general SDM than HC ($p < .01$ for both measures).<br>Both AD and HC produced more integrated than non-integrated memories (AD= $p < .05$; HC= $p < .001$)<br>No significant differences between AD and HC were reported in ratings of centrality ($p > .10$) or self-continuity ($p > .10$) of SDM<br><br>Both AD and HC produced more positive than neutral memories (AD= $p < .01$; HC=$p < .001$) and more positive than negative memories (AD= $p < .05$; HC= $p < .05$).
**El Haj et al., 2012**	To examine the involuntary nature of music-evoked AM in AD compared to healthy older and younger adults.	16 patients with probable AD<br>Mean age: 75.94 (SD 6.21)<br>Sex: F and M<br><br>16 Older control	TEMPau test [Piolino et al., 2000; 2006]	Recalling autobiographical memories in two conditions:<br>-Silence condition<br>-Music condition (listening to	AD patients produced less specific memories than both older control subjects ($p < .05$), and younger control subjects ($p < .05$).<br>AD patients produced more specific memories in the Music condition($p < .05$).<br>Music-evoked memories had more positive emotional content in older control subjects ($p < .01$)<br>AD patients reported a mood enhancement in the Music condition($p <.05$).<br>AD reported a larger reduction of retrieval time ($p < .05$) compared to older control subjects ($p < .05$) or younger control subjects ($p < .05$).

		subjects<br>Mean age: 73.75 (SD 6.54)<br>Sex: F and M<br><br>16 Younger control subjects<br>Mean age: 21.19 (SD 2.88)<br>Sex: F and M		self-chosen music)	
**El Haj et al., 2013**	To investigate how music exposure affects the linguistic characteristics of AM in AD	18 patients with probable AD<br>Mean age: 75.83 (SD 5.85)<br>Sex: F and M<br><br>18 control subjects<br>Mean age: 73.61 (SD 6.27)<br>Sex: F and M	TEMPau test [Piolino et al., 2006; Piolino, Desgranges, & Eustache, 2000]	Recalling in detail an event in your life" in two conditions:<br>-After 2 minutes of silence<br>-After 2 minutes of listening to their own chosen music	AD showed significant improvement in autobiographical recall after music exposure compared to silence condition ($p < .01$).<br>Music exposure in AD patients led to:<br>-Fewer empty words ($p < .001$)<br>-Increased grammatical complexity ($p < .001$)<br>-Increased propositional density ($p < .001$)

**El Haj et al., 2015 Self-defining**	To investigate whether exposure to music could enhance the production of self-defining memories in AD	22 patients with probable AD Mean age: 71.73 (SD 6.99) Sex: F and M<br><br>24 control subjects Mean ag: 72.88 (SD 7.34) Sex: F and M	adaptation of the TEMPau scale [Piolino et al., 2006; 2007]	Recalling in detail, an event in your life" for 3 minutes during exposure to (1) self-chosen music, (2) researcher-chosen music, and (3) in silence.	AD evoked more self-defining memories when exposed to their own-chosen music than when exposed to researcher-chosen music or in silence ($p < 0.01$ for both comparisons).<br>AD patients reported greater autobiographical recall during exposure to their own-chosen music than to researcher-chosen music ($p < 0.05$), during exposure to researcher-chosen music than in silence ($p < 0.05$), and during exposure to their own-chosen music than in silence ($p < 0.01$). AD evoked more self-defining memories than autobiographical-episodes or personal-semantics during exposure to their own-chosen music ($p < 0.05$ for both comparisons).<br>AD produced fewer self-defining memories than HC in silence ($p < 0.05$) and during exposure to researcher-chosen music ($p < 0.05$), but no significant differences were observed during exposure to self-chosen music ($p > 0.10$).<br>HC did not show significant differences in self-defining memories production across the three experimental conditions.
**El Haj et al., 2015 Similarity**	To explore the similarities between past and future autobiographical generation in AD.	27 patients with probable AD Mean age: 71.85 (SD 7.01) Sex: F and M<br><br>30 control subjects Mean age 72.47 (SD 7.04) Sex: F and M	TEMPau scale (Test épisodique de mémoire du passé) [Piolino et al., 2000, 2006, 2007]	Recalling in detail an event in their lives or imagining a future even providing details and describing the time and place at which events had/will have occurred, as well as to their feelings and	Significantly more individuals with AD repeated the same themes when recalling past events and imagining future events, compared to HC ($p<0.05$).<br><br>Participants with AD generated significantly fewer self-defining memories than HC, both when recalling past events ($p<0.05$) and when imagining future events ($p<0.001$).

				emotions during those events.	
**El Haj et al., 2015**<br><br>**Flexibility**	To examine the role of executive functions in past and future thinking in AD patients.	24 patients with probable AD<br>Mean age: 72.08 (SD 7.20)<br>Sex: F and M<br><br>26 control subjects<br>Mean age: 72.58 (SD 7.20)<br>Sex: F and M	TEMPau scale [Piolino et al., 2003, 2006, 2007]	Recalling a past event and imagine a future event (3 minutes each)	AD patients reported worse autobiographical performance during past thinking (p=0.01) and during future thinking (P=0.01).<br>AD and HC recalled similar autobiographical performance for past and future events.<br>Cognitive flexibility correlated and predicted the similarity between past and future thinking in both AD and HC(p < 0.01).<br><br>AD patients with lower flexibility showed higher similarity between past and future thinking compared to those with higher flexibility (p = 0.01)<br>In AD, future thinking was significantly correlated with binding ability (p < 0.05)
**El Haj et al., 2016**	To investigate various phenomenological features of AM and determine their relative vulnerability in AD	27 patients with probable AD= 27;<br>Mean age: 71.41 (SD 5.46)<br>Sex: F and M<br><br>30 control subjects<br>Mean age =	TEMPau scale [Piolino et al., 2000; 2006]<br><br>Autobiographical Memory Questionnaire (AMQ) [Rubin et al., 2003]	Recalling in detail an event in your life" and given 5 minutes to describe their memories.	AD showed poorer general autobiographical recall compared to control subjects (p < .01).<br>AD demonstrated lower ratings for reliving (p < .05), travel in time (p < .05), remembering (p < .01), realness (p < .01), visual imagery (p < .001), auditory imagery (p < .01), language (p < .05), rehearsal (p < .01), spatial details (p < .001) and temporal details (p < .01) compared to control subjects.<br>No significant differences were found between groups for emotion and importance ratings (p > .1).<br>Visual imagery showed the most pronounced decrease in AD compared to other phenomenological features. Despite overall decreases, AD showed high ratings for emotion and importance.

		68.73 years (7.84)<br>Sex: F and M			
**El Haj et al., 2017**	To investigate whether retrieving information related to conceptual self would improve autobiographical memory in AD.	24 patients with probable AD<br>Mean age: 71.20 (SD 5.29)<br>Sex: F and M<br><br>27 control subjects<br>Mean age: 68.41 (SD 8.17)<br>Sex: F and M	TEMPau scale [Piolino et al., 2002]	Recalling in detail an event in their life after either generating "Who am I?" statements or reading a control text.	AD patients generated fewer "Who am I?" statements compared to HC ($p < .001$).<br>AD showed higher autobiographical specificity after "Who am I?" statements than after text reading ($p < .05$).<br>AD showed more context recall after "Who am I?" statements than after text reading ($p < .05$)<br>AD reported higher relieving after "Who am I?" statements than after text reading ($p < .01$).
**El Haj et al., 2017 Discrepancy**	To determine a possible discrepancy between subjective and objective autobiographical recall in AD.	31 patients with probable AD<br>Mean age: 71.76 (SD 5.38)<br>Sex: F and M<br><br>35 control subjects<br>Mean age: 69.29 (SD 7.54)	Subjective reliving:<br>10-item scale from the Autobiographical Memory Questionnaire [Rubin et al., 2003]<br><br>Objective recall:	Recalling three autobiographical memories cued by "family event", "professional event", and "holiday event".	AD patients showed poorer subjective ($p < 0.001$) and objective ($p < 0.001$) reliving than HC.<br>IN AD patients there was a higher discrepancy between subjective and objective reliving (higher subjective ratings compared to objective performance) compared to HC ($p < 0.001$). This discrepancy was significantly greater in AD compared to HC ($p < 0.01$).

		Sex: F and M	TEMPau scale [Piolino et al., 2002]		
**El Haj et al., 2018**	To examine the potential involuntary nature of odor-evoked and music-evoked AM in AD.	28 patients with probable AD Mean age: 73.25 years, (SD 6.71) Sex: F and M<br><br>30 control subjects Mean age: 71.75 (SD 8.05) Sex: F and M	TEMPau scale [Piolino et al. 2004, 2006, 2007; Rauchs et al. 2007]	Recalling personal memories in 3 conditions: after odor exposure, after music exposure, and in a control condition. Memories were scored for specificity, emotion, mental time travel, and retrieval time	AD participants showed higher specificity, emotional experience, mental time travel, and shorter retrieval time for memories after odor and music exposure compared to control condition ($p < 0.05$ for all)<br>Retrieval time was significantly shorter after odor exposure compared to music exposure in both AD and HC ($p < 0.001$)<br>Executive function predicted AM in the control condition but not after odor or music exposure in AD participants.
**El Haj et al., 2019 Mental imagery and autobiographical memory**	To investigate the relationship between AM and mental imagery abilities in AD.	26 patients with probable AD=26 Mean age 73.35 (SD 6.55) Sex: F and M<br><br>28 control	TEMPau scale [Piolino et al., 2006]	Recalling two autobiographical events and providing detailed descriptions. These were scored on a 0-4 point scale for	AD showed impaired AM compared to HC ($p < .001$)<br>AD showed preserved visual imagery but lower spatial imagery compared to HC ($p < .001$ for spatial imagery)<br>Significant correlations were found between AM and both visual and spatial imagery in AD and HC ($p < .01$)<br>Visual imagery significantly predicted autobiographical memory performance in regression analyses for both AD and HC

		subjects<br>Mean age<br>70.67 (SD 8.99)<br>Sex: F and M		specificity and detail.	
**El Haj et al., 2019**<br><br>**Autobiographical recall**	To examine if recalling personal memories could enhance the sense of self in individuals with AD.	28 patients with probable AD<br>Mean age: 71.14 (SD 4.56)<br>Sex: F and M<br><br>31 control subjects<br>Mean age: 68.42 years (7.67)<br>Sex: F and M	TEMPau scale [Piolino et al., 2002]	Recalling in detail an event in their life and describing their memories associated Responses were scored on a 0-4 scale for specificity	AD patients showed lower autobiographical specificity compared to HC ($p < .001$)<br>This effect was seen across all three self-dimensions: Physical self: AD $p < .05$<br>Social self:<br>AD, $p < .01$, Psychological self:<br>AD $p < .05$,
**El Haj et al., 2019**<br><br>**Memories Supporting Myself:**	To investigate how patients with mild AD reflect on continuity of their self by means of AM.	32 patients with probable AD<br>Mean age 73.25 (SD 6.56)<br>Sex: F and M<br><br>35 control subjects<br>Mean age	The Thinking about Life Experiences (TALE) Scale [Bluck et al., 2005]	Recalling events and memories and reflecting on the connection of these events and the present on a five-point Likert-type	AD mean score on the TALE Scale was not significantly different from HC score ($p > 0.10$).<br>AD scored significantly higher than HC on Item 1: "When I want to feel that I am the same person that I was before" ($p < 0.01$)<br>AD showed significantly higher scores on Item 1 compared to other items (all $p < 0.01$)

		70.31 (SD 8.26)<br>Sex: F and M		scale, with one = almost never and five = very frequently.	
**El Haj et al., 2020 Memory of decisions**	To examine temporal discounting in AD and investigate its relationship with decline in autobiographical memoryAM, executive function, and general cognitive decline.	37 patients with probable AD=37<br>Mean age 70.3 (SD 7.02)<br>Sex: F and M<br><br>40 control subjects<br>Mean age 69.28 (SD 5.56)<br>Sex: F and M	TEMPau scale [Piolino et al., 2002]	Recalling three autobiographical memories, each cued with the instruction "recount in detail an event in your life." They had 2 minutes for each memory.	AD participants showed lower scores in inhibition (p < .001), in shifting (p = .015) and autobiographical memory (p < .001).<br>AD showed higher temporal discounting than HC (p = .002)<br>AD reported significant negative correlations between temporal discounting and AM scores (p = .004) and significant negative correlations between temporal discounting and general cognitive functioning (r = -.50, p = .001).
**El Haj et al., 2020 The past**	To examine sex differences in AM in patients with AD and in control subjects in terms of	32 patients with probable AD<br>Mean age: 71.28 (SD 4.68)<br>Sex: F and M	TEMPau scale [Piolino et al., 2002]	Recalling two autobiographical memories and to provide spatiotemporal details	lower specificity was observed in AD than in HC (P<0.001) lower time travel was observed in AD than in HC (P<0.001) lower visual imagery was observed in AD than in HC (P<0.001) slower retrieval time was observed in AD than in HC (P<0.001).

	specificity, subjective experience (ie, mental time travel, emotion, and visual imagery), and retrieval time	35 control subjects<br>Mean age: 69.80<br>(SD 6.46)<br>Sex: F and M			
**El Haj et al., 2020**<br><br>**la vie en rose**	To examine the emotional regulation of AM in AD.	28 patients with probable AD<br>Mean age: 71.71 (SD 5.12)<br>Sex: F and M<br><br>30 control subjects<br>Mean age: 68.80 (SD 8.01)<br>Sex: F and M	TEMPau scale [Piolino et al., 2002]<br><br>AMI [Kopelman et al., 1990]	Recalling a personal event in response to the cues "happy" and "sad". Rating the emotional valence of the memory at retrieval and encoding on a 7-point scale.	AD showed lower autobiographical specificity than HC for both "happy" ($p < .001$) and "sad" memories ($p = .001$).<br>Both AD and HC rated "happy" memories as more positive at retrieval than encoding ($p < .01$).<br>Both AD and HC rated "sad" memories as less negative at retrieval than encoding ($p < .001$).<br>Emotional regulation was negatively correlated with depression scores in both AD and HC ($p < .05$).
**El Haj et al., 2020**<br>**The picture of the past**	To investigate the effect of pictorial cues on AM in AD.	27 patients with probable AD<br>Mean age: 71.67 (SD 5.31)<br>Sex: F and M<br><br>30 Control subjects	TEMPau scale [Piolino et al., 2002]	Recalling three autobiographical memories in each condition in no cue condition (1 condition) and verbal	AD participants showed lower autobiographical specificitypiolino during the no cue condition ($p < .001$) and during the verbal-and-visual cuing condition ($p < .001$).<br>AD and HC showed higher autobiographical specificity in the verbal-and-visual cuing condition compared to the verbal-only condition: AD=$p < .01$, HC=$p < .01$.<br>In AD patients there was a significant positive correlation between scores on the Grober and Buschke test and autobiographical performances in the no cue condition ($p = .018$) and verbal-and-visual cuing condition ($p = .029$).

		Mean age: 68.80 (8.01)<br>Sex: F and M		and visual cuing simultaneously (2 condition).	
**El Haj et al., 2022**<br>**The fabricated past**	To investigate intentionally fabricated autobiographical memories in AD.	AD=25;<br>Mean age: 72.44 years (6.87)<br>Sex: F and M<br><br>HC= 28;<br>Mean age: 73.29 years (7.75)<br>Sex: F and M	Retrieval time: measured in seconds from the end of instructions to the start of memory construction<br><br>Vividness: rated on a five-point scale (1 = "no image at all" to 5 = "perfectly clear and as vivid as normal vision")	To generate one truthful autobiographical memory and one fabricated event that had never occurred. They were instructed to describe these events in detail.	Retrieval time:<br>Slower for fabricated memories than real ones in both AD ($p < .01$) and HC ($p < .01$)<br><br>AD had slower retrieval times than HC for both fabricated ($p < .001$) and real memories ($p < .001$)<br><br>Vividness:<br>AD: Similar vividness for fabricated and real memories ($p > .1$)<br><br>HC=Lower vividness for fabricated memories compared to real ones ($p < .05$)<br><br>AD showed lower vividness than controls for both fabricated ($p < .01$) and real memories ($p < .001$)
**El Haj et al., 2024**	To investigate the ability of patients with	41 patients with probable AD	the Sentence Completion for Events	Completing sentences with	AD produced fewer specific memories ($p < .001$), categoric memories ($p < .001$), and extended memories ($p = .001$), but more semantic memories ($p = .025$,) and omissions ($p < .001$).

	AD to construct specific AM using the Sentence Completion for Events from the Past Test (SCEPT)	Mean age: 70.32 (SD 3.24)<br>Sex: F and M<br><br>44 control subjects<br>Mean age: 70.11 (SD 3.88)<br>Sex: F and M	from the Past Test (SCEPT) [Raes et al. 2005]<br><br>Memories were categorized into specific, categoric, extended, or semantic memories.	autobiographical memories each probing for thoughts relating to the past (e. g., "When I think back to/of..., I still recall how/that I...")	
**El Haj, 2011**	To investigate intentional inhibitory processes in episodic and semantic autobiographical memory in AD using the directed forgetting method.	16 patients with probable AD<br>Mean age: 76.44 (SD 5.75)<br>Sex: F and M<br><br>16 control subject<br>Mean age: 74.38 (SD 4.22)<br>Sex: F and M	Adaptation of the autobiographical directed forgetting method [Barnier et al. 2007]<br><br>Distinction between episodic and semantic autobiographical memories based on the TEMPau task criteria	Generating autobiographical memories in response to word cues from two lists. After List 1, they were instructed to either forget or continue remembering those memories. They then recalled memories from both lists regardless of instructions.	AD patients generated significantly fewer autobiographical memories than HC ($p <0.001$).<br>AD recalled fewer episodic than semantic memories overall ($p < .001$)<br>In AD no directed forgetting effects (List 1 costs or List 2 benefits) for either episodic or semantic memories ($p > .05$) were found.<br>No List 2 benefits observed in either group

**Eustache et al., 2004**	To investigate the neural substrates of autobiographical amnesia in AD using PET imaging and a correlative approach between resting cerebral glucose utilization and temporally graded memory scores	17 patients with probable AD<br>Mean age: 72.8 (SD 5.2)<br>Sex: F and M<br><br>14 control subjects<br>Mean age: 71.6 (SD 4.9)<br>Sex: F and M	A novel personal event task derived from AMI [Kopelman et al., 1989 Borrini et al. 1989 and Piolino et al. 2003]	Recalling specific detailed personal across three distinct time periods in an individual's life:<br>-Previous 5 years<br>-Middle age<br>-Teenage and childhood<br><br>Re-test conducted after 15 days to check memories	AD patients performed worse than controls across all time periods ($p < 0.0001$). AD patients showed a temporal gradient, with better preservation of remote memories($p < 0.0001$).<br>PET analysis showed correlations between autobiographical scores and metabolism in: -Right hippocampus for Period A<br>-Bilateral prefrontal cortex for -Period B<br>Left prefrontal cortex for Period C ($p < 0.01$)
**Fromholt et al., 2003**	To examine AM in centenarians compared to 80-year-old control, depressed, and dementia groups.	30 patients with AD<br>Mean age: 80.5<br><br>15 Centenarians subjects<br>Mean age: 100<br>Sex: F and M<br><br>30 control	Life narrative interview [Fromholt & Larsen, 1991]<br><br>Word-cued autobiographical memory task (for centenarians only in Experiment 2)	Experiment 1: Freely recalling memories for 15-minute<br><br>Experiment 2: Recalling memories in response to 15 cue words	Experiment 1<br>AD produced significantly fewer autobiographical memories compared to HC ($p < .01$) but a similar number to centenarians and the depressed patients.<br>AD patients recalled memories containing fewer details compared to HC ($p < .05$) but were similar in terms of detail to those of centenarians and the depressed group.<br>AD was the only one to show frequent repetitions of already told memories ($p < .05$)<br>AD had significantly more undated memories compared to the other groups ($p < .01$).<br><br>Experiment 2:<br>Despite cognitive difficulties, AD still showed a distribution of autobiographical

		subjects<br>Mean age: 78.3<br>Sex: F and M<br><br>15 patients with depression<br>Mean age: 80.2<br>Sex: F and M			memories similar to other groups, with childhood amnesia, reminiscence bump, and recency effects (p < .001).<br><br>World War II memories: Only 2 out of 30 AD spontaneously mentioned World War II events in their life narratives, compared to 10 out of 30 in the control group.
**Fromholt, P. et al., (1995).**	To investigate the effects of depression on autobiographical memory in older adults suffering from major depression for the first time, and to examine changes after recovery.	30 patients with AD<br>Mean age: 80.5 (SD 4.36)<br>Sex: F and M<br><br>15 Depressed patients (DP)<br>Mean age: 80.2 years (SD 5.27)<br>Sex: F and M<br><br>30 control subjects<br>Mean age: 78.3 (SD 4.81)	Free narrative method (Fromholt & Larsen, 1991)	Describing "events that have been important in your life" as a free narrative.	AD patients recalled fewer memories and details than HC, but similar to DP (p < .001).<br>AD patients present significantly higher undated memories than other groups (p < .001).<br>AD patients also showed significant prevalence of chronologically ordered event pairs (58.7% forward vs 23.6% backward, p < .001).

		Sex: F and M			
**Genon, S. et al., 2014**	To examine how self and memory processes interact in people with AD compared to healthy elderly adults.	21 patients with probable AD Mean age: 76.1 (SD 6.0) Sex: F and M<br><br>21 control subjects Mean age 76.0 (4.9) Sex: F and M	No direct measures of autobiographical memory were used. The study focused on the self-reference effect (SRE) on episodic memory<br><br>procedure derived from the traditional Remember/Know procedure (Tulving, 1985; Gardiner, 1988).	Self-recognition task: encoding of adjectives in reference to self or others, followed by a yes/no recognition task. Self-recollection task: similar to the first, but with a modified Remember/Know procedure during recognition.	Self_accuracy and Other_accuracy were significantly lower in AD than HC group (both ps < .001).<br>Self_R and Other_R were significantly lower in AD group than HC group (both ps < .001)<br>AD patients showed significantly higher rates of false remembered responses compared to HC, both for self-referenced and other-referenced items (both ps < .001).
**Glachet & El Haj, 2022.**	To investigate whether odor exposure could enhance access to self-concept in	24 patients with probable AD Mean age: 85.12 (SD 5.68)	The production of self-related statements ("Who am I?") [Charlesworth et al., 2016] in	Producing self-related statements in response to the question "Who am I?"	AD patients produced fewer "Who am I?" statements than HC in both conditions (p < .001 for odor-free condition, p = .003 for odor condition).<br>Both AD and HC produced more total "Who am I?" statements in the odor condition compared to the odor-free condition (AD: p < .001, HC: p = .025).<br>AD produced more psychological self-statements in the odor condition compared to the odor-free condition (p = .034).There was no significant difference in social or

	AD patients.	Sex: F and M<br><br>25 control subjects<br>Mean age: 84 (SD 8.5)<br>Sex: F and M	odor and odor-free conditions		physical self-statements between odor and odor-free conditions for AD and HC.
**Glachet & El Haj., 2021**	To compare the effectiveness of odor cues versus visual cues and verbal cues for triggering AM in AD	23 patients with probable<br>Mean age: 84.87 (SD 5.67)<br>Sex: F and M<br><br>24 control subjects<br>Mean age: 84.33 (SD 8.52)<br>Sex: F and M	TEMPau scale [Piolino et al., 2002]<br><br>-Specificity<br>-Subjective experience<br>-Emotional characteristics<br>-Retrieval time	Recalling and describing an AM after being presented with an odor cue, visual cue, or verbal cue of the same item.	AD patients reported less specificity in the odor-cue condition $p < .001$), in the visual-cue condition ($p < .001$) and in the verbal-cue condition ($p < .001$) compared to control subjects.<br>Odor cues evoked more specific memories than visual or verbal cues in both AD and control subjects ($p < .001$ for AD, $p < .001$ for control subjects).<br>Odor-evoked memories were retrieved faster than those cued by visual or verbal stimuli in both groups ($p < .001$ for AD, $p < .001$ for HC)<br>Odor cues led to higher ratings of emotional intensity and more positive valence compared to other cues in both groups ($p < .001$ for intensity, $p < .001$ for valence in AD).<br>Odor cues enhanced several aspects of subjective experience compared to other cues, especially in AD
**Glachet et al., 2019 Emotional and Phenomenological**	To investigate the effects of odor exposure on the emotional qualities and	25 patients with AD<br>Mean age: 82.04 (SD 7.34)	TEMPau scale [Piolino et al., 2002]	Recalling autobiographical memories with special meanings in	In AD, odor-evoked memories showed:<br>Higher specificity ($p < 0.01$)<br>Greater subjective reliving ($p < 0.05$)<br>Higher arousal ($p < 0.01$)<br>More positive valence ($p < 0.01$) Compared to the odor-free condition

	subjective reliving of AM in AD and examine relationships with depression.	Sex: F and M<br><br>23 control subjects<br>Mean age: 80.91 (SD 9.87)<br>Sex: F and M		two conditions:<br>-After exposure to a cinnamon odor<br>-Without odor exposure (control condition)	Negative correlations were found in AD between depression scores and arousal ($r = 0.11$, $p > 0.05$), valence ($r = -0.44$), and subjective reliving ($r = −0.48$, $p < 0.05$) for odor-evoked memories.
**Glachet et al., 2019 Smell your memories**	To investigate the effects of odor exposure on the recall of recent and remote AM in AD.	26 patients with probable AD<br>Mean age: 72.69(SD 6.63)<br>Sex: F and M<br><br>28 control subjects<br>Mean age: 70.82 (SD 7.81)<br>Sex: F and M	Adaptation of the EAMI [Irish et al., 2008] , TEMPau task [Piolino et al., 2006, 2007], and the AMI [Kopelman, 1994].	Recalling two childhood memories, two adulthood memories, and two recent memories.	AD produced more childhood memories ($p < .05$), adulthood memories ($p < .01$), and recent memories ($p < .01$) after odor exposure compared to without odor.<br>In both conditions (with and without odor), AD recalled more adulthood episodes than childhood episodes ($p < .01$ for both), more adulthood episodes than recent episodes ($p < .01$ for both), and more childhood memories than recent memories ($p < .05$ for both).<br>HC showed no significant differences in memory retrieval between odor and no-odor conditions ($p > .1$ with odor, $p > .1$ without).
**Greene et al., 1995**	To test if frontal lobe-based	17 patients with probable	AMI [Kopelman et al., 1999]	Recalling events occurring	Patients with minimal AD ($p<0.0001$) and with mild AD ($p<0.0001$) performed significantly worse than HC, but that there was no difference between minimal and mild AD in autobiographical incident

	'executive' functions are vital for the retrieval of AM.	minimal AD Mean age: 73.1 (SD 8.2) Sex: F and M<br><br>16 patients with probable mild AD Mean age: 66.2 (SD 8.0) Sex: F and M<br><br>30 control subjects Mean age: 67.9 (SD 8.7) Sex: F and M		before the onset of the pathology (e.g. an incident occurring on holiday prior to onset of memory problems).	Patients with minimal AD (P<0.0001) and mild AD(P<0.0001) performed worse than HC in AMI, but there was no difference between patient groups (i.e. minimal=mild AD < HC)
**Greene et al., 1996**	To study remote memory (both autobiographical and public) longitudinally over a 1-year period in patients with AD compared to HC.	24 patients with AD; Mean age: 69.8 (SD 8.6) Sex: F and M<br><br>30 control subjects Mean age: 67.9 (SD 8.7) Sex: F and M	AMI [Kopelman et al., 1990]<br><br>Autobiographical Fluency Test [Dritschel et al., 1992]	AMI= Recalling personal semantic and autobiographical incident memory across three time periods (childhood, early adulthood, recent life)<br><br>Autobiographical	AD patients reported memory impairments both in the AMI and autobiographical fluency tests compared to control subjects at baseline ($p < 0.001$ for all measures). Public memory (famous face and name identification) deteriorated significantly over 1 year in AD patients ($p < 0.001$)<br>Autobiographical memory did not show significant deterioration over 1 year in AD patients ($p > 0.05$ for all autobiographical measures)

				Fluency=Producing names and incidents for childhood, early adulthood and late adulthood in 90 seconds each	
**Greene et al.,1996 Neuropsychology of memory**	To compare the usefulness of neuropsychological testing vs SPECT imaging for diagnosis and staging of AD.	33 patients with AD Mean age: 69.9 (SD 8.6)<br><br>30 control subjects for Neuropsychological tests Mean age 67.9 (SD 8.7)<br><br>24 control subjects for SPECT imaging Mean age: 65.4 (SD 8.2)	AMI [Kopelman et al., 1999]<br><br>Autobiographical fluency test [Dritschel et al., 1997]	AMI: Recalling personal semantic and autobiographical incident memory across 3 time periods<br><br>Autobiographical fluency: Generating names and incidents from 3 time periods in 90 seconds each	AD patients performed significantly worse than the control subjects across all components, with p-values less than 0.001 in all cases<br>For SPECT imaging, differences in regional cerebral blood flow between AD and controls were significant for:<br>Right frontal ($p < 0.001$), Right high frontal ($p < 0.001$), Right temporal ($p < 0.01$), Right parietal ($p < 0.05$), Right posterior temporal ($p < 0.05$) and Left frontal ($p < 0.05$).

**Han et al., 2014**	To examine vocal expression of emotions (EE) in the retrieval of AM in early stage AD.	21 patients with AD Mean age 61.84 (SD 5.2)<br>Sex: F and M<br><br>HC=19; Mean age 61.84 (SD 3.74)<br>Sex: F and M	AI [Levine et al., 2002] including four categories of retrieval: recent (yesterday, last week, last month) and remote memories	Freely recalling impressive events from different time periods.<br><br>For remote memory, subjects recalled impressive event from childhood; for recent memory they retrieved any impressive event of the last month, last week, and yesterday.	AD patients showed lower EE in both recent and remote memories compared to HC ($p < 0.0001$ for all memory categories)<br><br>Both AD and HC reported a higher emotional involvement for remote memories compared to recent memories (AD: $p < 0.001$; HC: $p < 0.0039$)<br>Positive correlations were found between EE and cognitive functions.
**Hirjak et al., 2017**	To examine the relationship between hippocampal and extrahippocampal alterations	38 patients with probable AD Mean age: 73.63 (SD 5.82)	E-AGI (Fast et al., 2007)	Recalling semantic memories (SEM). Recalling autobiographi	AD and MCI showed relatively well-preserved SEM for childhood and adulthood periods while AD showed significant impairment for the recent 5 years compared to MCI and HC ($p < 0.001$)<br>AD showed significant impairment in EP-F and EP-F across all time periods compared to HC and MCI ($p < 0.001$).<br>MCI showed intermediate performance, significantly different from both AD and HC

	and AM deficits in AD and MCI.	Sex: F and M<br><br>38 patients with MCI<br>Mean age: 72.71 (SD 6.62)<br>Sex: F and M<br><br>31 control subjects<br>Mean age: 71.13 (SD 4.46)<br>Sex: F and M		cal incident (free recall of autobiographical episodes -EP-F and rating according to uniqueness of narrative statements and richness of details -EP-D).	for adulthood and recent 5 years ($p < 0.001$).
**Hou et al., 2005**	To examine the dissociation of memory for autobiographical incidents and personal semantic information in patients with AD, frontotemporal dementia (FTD), and SD.	9 patients with probable AD<br>Mean age: 74.5 (SD 9.6)<br><br>11 patients with FTD<br>Mean age: 62.2 (SD 11.5)<br><br>8 patients with SD<br>Mean age: 63.4 (SD 9.3)<br><br>8 control	AMI [Kopelman et al., 1989]	Recalling autobiographical incidents and personal semantic information from three time periods: childhood, early adulthood, and recent life.	AD patients reported no significant gradient effect for autobiographical incidents or personal semantic information<br>AD patients performed worse than other groups in recalling recent autobiographical incidents and personal semantic information ($p < 0.05$).<br>SD patients recalled more autobiographical incidents from recent life than childhood ($p < 0.01$) and early adulthood ($p < 0.01$). Showed a gradient effect for personal semantic information, with better recall from more recent time periods (recent life vs early adulthood: $p < 0.001$; early adulthood vs childhood: $p < 0.05$)FTD patients reported no significant difference in recall across time periods for either autobiographical incidents or personal semantic information.

		subjects<br>Mean age: 70.7 (SD 6.3)			
**Irish et al., 2006**	To examine the enhancing effect of music on AM recall in individuals with mild AD.	10 patients with probable AD<br>Mean age 76.3 (SD 7.48)<br>Sex: F and M<br><br>10 control subjects<br>Mean age 76.5 (SD 5.19)<br>Sex: F and M	AMI [Kopelman et al., 1991]	Recalling memories over three life periods: childhood, early adult life, and recent life. Answering questions related to major life events such as education, employment, weddings, etc.	AD showed lower AMI scores compared to HC in both conditions (silence: $p < 0.001$ music: $p < 0.005$).<br>AD patients reported significant improvement in AMI recall during music condition compared to silence condition ($p < 0.005$).<br>AD patients reported a temporal gradient from childhood through adulthood to recent memories.<br>AD showed a significant reduction in state anxiety during music condition ($p < 0.05$).
**Irish et al., 2011 Impaired capacity**	To investigate AM retrieval and autonoetic consciousness in AD and control subjects.	20 patients with probable AD<br>Mean age: 73.0 (SD 7.4)<br>Sex: F and M<br><br>30 middle aged control	EAMI [Irish et al., 2008]	Recalling 3 specific events from each of 5 life periods from Childhood (0–15 years), to Early Adulthood	AD patients recalled significantly less details across all life epochs compared to HC ($p < .0001$ for all epochs). AD patients reported a temporal gradient, recalling less contextual details for more recent epochs.<br>AD patients were more accurate in temporal specificity for Early Adulthood, with significant decline in subsequent epochs (e.g., Middle Adulthood $p < .0001$; Later Adulthood $p < .0001$; Recent period $p < .0001$).

		subjects<br>Mean age: 40.6 (SD 10.3)<br>Sex: F and M<br><br>30 healthy elderly controls;<br>Mean age: 73.2(5.2)<br>Sex: F and M		(16–30 years), to Middle Adulthood (31–45 years), to Later Adulthood (46 up to 5 years ago) to Recent memory (in the last 5 years).	
**Irish et al., 2011 Profiles**	To compare AM retrieval in AD, SD and bvFTD, focusing on the differential patterns of contextual details of recent period.	17 patients with AD<br>Mean age: 66.1 (SD 8.6)<br>Sex: F and M<br><br>25 patients with SD<br>Mean age: 62.6 (SD 8.1)<br>Sex: F and M<br><br>15 patients with bvFTD<br>Mean age: 61.6 (SD 7.4)	AI [Levine et al., 2002]<br><br>Internal (episodic) and external (semantic) details were scored<br><br>Details were further categorized into Event, Time, Place, Perceptual, and	Recalling detailed descriptions of first person experienced events from four different life epochs. including free recall, general probe, and specific probe conditions.	AD patients reported more external than internal details in the free recall condition ($p < .01$).<br>AD and bvFTD showed reduced capacity to recall specific and contextually rich ABMs across all life epochs ($p < .0001$ compared to control subjects) while SD showed relatively preserved recent AM compared to remote epochs ($p < .01$ for all comparisons).<br><br>In the recent period, AD patients reported impairments for free recall of Event ($p = .001$), Emotion/Thoughts ($p = .006$) and Perceptual ($p = .005$)<br><br>details.<br>SD were impaired in Spatiotemporal ($p < .0001$) and Emotion/Thoughts ($p < .0001$) details.<br>bvFTD were significantly impaired across all categories of contextual details (all ps < .05)<br>In recent probe recall AD patients showed significant deficits for recall of Event ($p = .001$) and Emotion/Thoughts ($p = .01$) details.

		Sex: F and M<br><br><br>19 control subjects<br>Mean age;<br>66.7 (SD 4.9)<br>Sex: F and M	Emotion/Thoughts		All patient groups showed impairment in the retrieval of Emotion/Thoughts ABM details (all ps < .01).
**Irish et al., 2012**	To examine the mechanisms and neural correlates of simulations in both episodic and semantic domains in patients with AD and Semantic Dementia (SD)	11 patients with AD<br>Mean age:<br>64.6 (SD 6.1)<br>Sex: F and M<br><br>11 patients with SD<br>Mean age:<br>62.1 (SD 5.5)<br>Sex: F and M<br><br>10 control subjects<br>Mean age:<br>69.2 (SD 5.6)<br>Sex: F and M	Autobiographical Interview (AI) [Levine et al., 2002]	Recalling specific events in their lives from the past year, and imagining possible events that could happen in their lives in the next year.	In the past condition, AD recalled significantly fewer past internal details than HC (p< 0.0001).<br>AD and SD were equally impaired relative to HC for future internal details.<br>AD performed equally poorly in the past and future conditions (p = 0.154).
**Irish et al., 2014**	To investigate the grey and white matter	15 patients with AD	AI [Levine et	Recalling memories	AD, bvFTD and SD reported significant impairments in remote memory retrieval compared to HC (AD, p = .001; bvFTD, p = .001; SD, p = .001) while SD patients

	correlates of recent and remote AM retrieval in AD, SD, in behavioural-variant frontotemporal dementia (bvFTD) and control subjects.	Mean age: 68.3 (SD 8.8)<br>Sex: F and M<br><br>11 patients with bvFTD<br>Mean age: 62.4 (SD 7.3)<br>Sex: F and M<br><br>10 patients with SD<br>Mean age: 64.0 (SD 8.9)<br>Sex: F and M<br><br>14 control subjects<br>Mean age: 72.2 (SD 3.9)<br>Sex: F and M	al., 2002]	from 4 life periods: Teenage Years, Early Adulthood, Middle Adulthood, and Recent period in Free Recall, General Probe, and Specific Probe conditions.	scored similarly to HC for recent retrieval (SD, p= .279; AD,p = .001; bvFTD, p = .007).<br>AD, SD, bvFTD and HC showed equivalent scores in AM across time periods (AD, p = .155; bvFTD, p= .548; Control, p = .288).<br>AD showed a decrease in grey matter intensity compared to HC specifically on the left hand side.
**Irish et al., 2016**	To examine the frequency of past tense usage in autobiographical memory narratives, comparing patients with AD, SD and control	10 patients with AD<br>Mean age: 65.5 (SD 6.1)<br>Sex: F and M<br><br>10 patients with SD<br>Mean age: 61.5 (SD 4.4)<br>Sex: F and M	Modified AI [Levine et al., 2002]	Describing 3 personally relevant events from the past year in response to cue words	Both AD and SD groups showed reduced use of past tense verbs compared to HC (p=.002 for AD, p=.031 for SD)<br>AD had a more frequent use of present tense verbs compared to HC (p=.004)<br>Both AD and SD produced fewer internal/episodic verbs (SD, p = .014; AD, p = .003) and more external/semantic verbs compared to HC (AD, p = .001; SD, (p = .056).<br>Past tense use correlated with integrity of frontal-temporal regions in AD and temporal lobe regions in SD.

	subjects. .	10 control subjects<br>Mean age: 69.0 (SD 5.6)<br>Sex: F and M			
**Ivanoiu et al., 2006**	To investigate whether episodic and semantic AM would dissociate in AD and SD patients.	20 patients with probable AD<br>Mean age: 76 (SD 7)<br>Sex: F and M<br><br>2 patients with SD<br>Mild SD: 72-year-old male<br>Moderate SD: 80-year-old female<br><br>21 control subjects<br>Mean age: 67 (SD 7)<br>Sex: F and M	a cued component AMI [Kopelman et al., 1990]<br><br>a free recall part using autobiographical fluency method [Dritschel, Williams, Baddeley, & Nimmo-Smith , 1992]	Cued and freely recalling of both episodic and semantic autobiographical memories across four life periods.	AD showed impairment in both episodic and semantic AM, with episodic memory more severely affected compared to control subjects ($p < 0.0001$).<br>Episodic AM was more severely impaired than semantic AM in AD patients ($p = 0.03$).<br>A temporal gradient was found for semantic but not episodic autobiographical memory in AD ($p = 0.0046$ for semantic, not significant for episodic).<br>AD recalled fewer "true" episodic memories compared to HC ($p = 0.0001$).<br>No significant correlations were found between autobiographical memory scores and executive function tests in AD<br><br>The mild SD patient showed preserved episodic autobiographical memory but impaired semantic autobiographical memory ($p < 0.0001$ for semantic impairment).<br>The moderate SD patient showed impairment in both episodic and semantic autobiographical memory ($p < 0.0001$ for both).

**Kazui et al., 2000**	To evaluate remote AM impairment in patients with AD	25 patients with probable AD<br>Mean age: 70.3 (SD 7.2)<br>Sex: F and M<br><br>25 control subjects<br>Mean age: 68.0 (SD 5.1)<br>Sex: F and M	the Family Line Test (FLT) .	Answering 20 questions about personal events across three lifetime periods: the subject's self, oldest child, and oldest grandchild.	AD patients reported significantly lower total FLT score compared to controls ($p < 0.001$).<br>A significant temporal gradient in remote memory impairment was observed in AD patients ($p < 0.001$).<br>Duration of remote memory impairment was significantly longer in AD patients compared to controls ($p < 0.001$).<br>FLT scores correlated significantly with general cognitive function and recent memory performance (p values ranging from < 0.05 to < 0.001 for various cognitive measures).<br>AD patients showed worse performance in more recent lifetime periods (oldest grandchild < oldest child < subject's self, $p < 0.001$ for each comparison).
**Kirk et al., 2018**	To investigate whether concrete objects historically dated to participants' youth allow easier access to AM in AD patients compared to verbal cues.	49 participants with AD<br>Mean age: 80.47 (SD 7.29)<br>Sex: F and M<br><br>50 control subjects<br>Mean age 81.20 (SD 6.49)<br>Sex: F and M	A novel version of an autobiographical task [Crovitz and Schiffman, 1974]	Being exposed to 5 concrete objects and 5 verbal cues historically dated to youth and recalling a personal memory in response to each cue.	AD and HC recalled significantly more memories when cued by objects vs words ($p < .001$) but the advantage was larger for AD ($p < .001$).<br>AD and HC reported higher episodic content scores when cued by objects vs words ($p < .001$).<br>AD and HC had longer descriptions for object-cued vs word-cued memories($p = .022$).<br>Patients with AD benefited from object-cueing regardless of cognitive impairment ($p < .001$).
**Leyhe et al., 2009**	To compare autobiographical memory among patients with AD, aMCI and	20 patients with probable AD<br>Mean age: 76.9 (SD 5.5)	AMI [Kopelman et al., 1999]	Recalling semantic personal information and episodic	AD showed impaired semantic and episodic autobiographical memory across all time periods compared to HC ($p < 0.001$).<br>AD exhibited a temporal gradient, with better preservation of childhood memories ($p < 0.01$).<br>aMCI showed impaired semantic and episodic autobiographical memory only for

	HC, examining both semantic and episodic components and potential temporal gradients.	Sex: F and M<br><br>20 patients with aMCI<br>Mean age: 72.6 (SD 6.8)<br>Sex: F and M<br><br>20 control subjects<br>Mean age: 71.6 (SD 6.5)<br>Sex: F and M		incidents across three time periods: childhood, early adulthood, and recent life	recent life compared to HC($p < 0.05$).
**Liechti, C. et al., 2019**	To investigate the relationship between hippocampal morphometry and autobiographical memory performance for events with different retrieval frequencies in AD compared to HC.	24 patients with probable AD<br>Mean age: 72.4 (SD 3.9)<br>Sex: F and M<br><br>27 control subjects<br>Mean age: 71.5 years (SD 6.6)<br>Sex: F and M	Historic Event Test (HET) [Leyhe et al., 2010]	Recalling personal circumstances surrounding famous historic events from the past 60 years. Events were categorized by retrieval frequency (low, medium, high).	AD performed worse than HC on autobiographical memory across all retrieval frequency conditions ($p < 0.001$)<br>Main effect of retrieval frequency on memory performance in both groups ($p < 0.001$)<br>In AD hippocampal volume correlated with memory performance for both seldom ($r = 0.670$, $p < 0.001$) and frequently retrieved events ($r = 0.549$, $p < 0.05$), with no significant difference between correlations ($z = 0.877$, n.s.)
**Lopis et al., 2021**	To define the	60 patients	A modified	Being	In AD patients pictures and odors were more effective than sounds in evoking

	most relevant sensory cue (odors, sounds or pictures) for AM retrieval in patients with AD.	with probable AD=60;<br>Mean age: 80.9 (SD 6.1)<br>Sex: F and M<br><br>60 older control subjects<br>Mean age: 80.1 (SD 6.2)<br><br>60 younger control subjects<br>Mean age: 22.2 (SD 2.9)	version of AM exposure task [Herz et al., (2004]	presented with either 4 odors, 4 sounds, or 4 pictures.<br>Recalling a particular person,place or event for each item presented.<br>Rating and dating the memories.	memories (p = 0.008). Sounds evoked significantly fewer memories than expected (p < 0.05). AD and older control subjects adults rated their memories as more emotional than younger control adults (p = 0.01 and p = 0.02 respectively).<br>AD recalled more memories from childhood/adolescence compared to older adults (p < 0.05).<br><br>Pictures were the most effective cues overall, helping to retrieve a higher number of memories (p < 0.05) that were also rarer (p = 0.03) across all groups.
**Martinelli et al., 2013**	To examine how normal aging and AD affect the recall of self-defining memories (SDMs) in comparison to typical autobiographical events (AE) and personal semantic	10 patients with probable<br>Mean age: 76.30 (SD 4.01)<br>Sex: F and M<br><br>18 younger control subjects<br>Mean age: 22.16 (SD	Tennessee Self-Concept Scale (TSCS) [Fitts & Warren, 1996: translated into French and validated in aging by Duval et al., 2007].	Recalling 10 AEs, 10 PSs, and 10 SDMs based on cue words.<br>Memories were scored on a 9-point episodic scale.	AD showed deficits in AE and SDMe compared to both younger and older control subjects(p < .001).<br><br>Older control subjects performed worse than the younger in AE (p < .001) but not in SDMe.<br>AD patients and older control subjects recalled a lower number of AE than PS and SDM (p .01 and p .001). In addition, the AD group recalled less SDM than PS (p .05)<br>AD patients and older control subjects recalled a higher number of positive PS compared to younger control subjects (p < .01).<br>AD patients showed no correlation between AM measures and self-concept.

	information (PS), and to explore the relationship between these memories and one's sense of self.	1.92)<br>Sex: F and M<br><br>16 older control subjects<br>Mean age = 75.18 (SD 4.61)<br>Sex: F and M			
**Meeter et al., 2005**	To investigate retrograde amnesia for semantic information in patients with AD and in control subjects.	16 patients with probable AD=16;<br>Mean age: 75.3<br>Sex: F and M<br><br>15 control subjects<br>Mean age: 76.3<br>Sex: F and M	Neologism and Vocabulary Test (NVT)<br><br>the Dutch adaptation of the AMI [Kopelman et al., 1989, 1990; Meeter & Murre, 2003]	Recalling anecdotes from different periods of their life (childhood, young adulthood, recent time periods).	AD performed worse than HC on the NVT ($p < .001$), indicating retrograde amnesia for semantic information.<br>AD patients reported stronger impairments in more recent periods in the AMI (p .026).<br>AD' performance on the NVT correlated with their performance on the National Adult Reading Test (NART) ($p < .05$), suggesting a link between semantic memory deficits and premorbid IQ estimates.<br>In the HC, NVT performance correlated with AMI performance ($p < .05$), indicating a relationship between semantic and episodic autobiographical memory
**Meléndez et al., 2016**	To compare episodic and	12 patients with AD	AMI [Kopelman et	Answering questions	AD reported lower scores in semantic memory compared to HC ($P < 0.001$) and the aMCI ($P = 0.021$).

	semantic AM across three life periods in AD, aMCI and healthy older adults.	Mean age: 83.1 (SD 9.5)<br>Sex: F and M<br><br>15 patients with aMCI<br>Mean age: 81.8 (SD 7.8)<br>Sex: F and M<br><br>29 control subjects<br>Mean age 78.2 (SD 5.1)<br>Sex: F and M	al., 1990]	about personal semantic information (e.g. name of the first school or teacher, first job, date and place of wedding, holidays or journeys). and autobiographical incidents from three time periods: childhood, early adult life, and recent life.	AD showed lower scores also in recent life memories compared both to HC and aMCI (P < 0.001).<br>AD reported lower scores on on episodic memory for all time periods (p < .001)<br>AD scores for recent life were significantly lower than the scores for the other two stages (P < 0.001), in both episodic and semantic memories.<br>No differences between aMCI and AD on episodic memory, but both lower than HC (p < .001).
**Meléndez et al., 2019**	To compare AM in AD, MCI and HC in order to examine differences in type of memory and emotional valence.	31 patients with AD<br>Mean age: 76.96 (SD 5.10)<br>Sex: F and M<br><br>32 patients with MCI<br>Mean age: 76.50 (SD 5.44)	AMT [Williams & Broadbent, 1986]<br><br>Responses coded for specificity (specific, general, vague) and emotional	Being exposed to 10 cue words (5 positive, 5 negative) and recalling a specific memory in response to each cue.	The AD group reported less specific memories compared to HC and MCI (AD, p < .05).<br>The AD group reported a greater number of vague responses (p < .001) than general and specific responses.<br>AD patients showed less positive memories than HC (p = .035).<br>HC and MCI showed more negative memories than AD (p < .005)<br>AD showed more neutral memories than HC and MCI (p < .003).

		Sex: F and M<br><br>32 control subjects<br>Mean age: 74.21 (SD 4.67)<br>Sex: F and M	valence		
**Meléndez et al., 2021.**	To examine how AM changes over time in patients with Alzheimer's disease (AD), patients with amnestic mild cognitive impairment (aMCI), and healthy control subjects (HC).	16 patients with AD<br>Mean age: 77.07 (SD 4.54)<br>Sex: F and M<br><br>17 patients with aMCI<br>Mean age:77.35 (SD 4.76)<br>Sex: F and M<br><br>26 control subjects<br>Mean age: 74.53 (SD 4.90)<br>Sex: F and M	Autobiographical Memory Interview (AMI) [Kopelman et al., 1999]	Recalling personal semantic content (involving retrieval of personal facts from one's past life) and autobiographical incidents (involving retrieval of episodes or incidents from one's past)<br>from three periods (childhood, early adult life, and	AD performed significantly worse than HC and aMCI at both baseline and follow-up in episodic AM ($p < 0.001$ for both time points).<br>AD showed significant decline in episodic AM over the 18-month period ($p < 0.001$).<br><br>At baseline, AD performed worse in semantic AD than both HC and aMCI ($p < 0.001$).<br>At follow-up, AD still performed worse than both other groups ($p < 0.001$).<br>AD showed significant decline in semantic AM over the 18-month period ($p < 0.001$).

				recent life).	
**Meulenbroek, O. et al., 2010**	To examine the neural underpinnings of autobiographical memory retrieval in AD patients compared to healthy elderly controls.	21 patients with probable AD<br>Mean age: 72.4 (SD 7.1)<br>Sex: F and M<br><br>22 control subjects<br>Mean age: 69.6 (SD 8.6)<br>Sex: F and M	AMI [Levine et al., 2002]<br><br>fMRI task involving true/false judgments on autobiographical and semantic statements	Evaluating the truthfulness of personalized autobiographical statements derived from their individual interviews	AD showed a shift from episodic to semantic elements in autobiographical memories compared to HC ($p < 0.001$)<br>During fMRI, AD showed enhanced activation in:<br>-Left inferior frontal gyrus ($p < 0.05$)<br>-Ventromedial prefrontal cortex ($p < 0.05$)<br>-Right precuneus ($p < 0.05$)<br>-Left lingual gyrus ($p < 0.05$)<br>In AD activation in ventromedial prefrontal cortex ($p < 0.05$) and left inferior frontal gyrus ($p < 0.05$) was negatively correlated with hippocampal volume.
**Moses et al., 2004**	To examine whether AD is associated with overgenerality of AM, specifically through an excess of categoric memories.	10 patients with possible<br>Mean age: 76.1 (SD 7.1)<br>Sex: F and M<br><br>10 control subjects<br>Mean age: 74.5 (SD 7.5)<br>Sex: F and M	Autobiographical Memory Test (AMT) [Williams & Broadbent, 1986]	Being presented with 12 cue words (6 positive, 6 neutral) and recalling a specific memory triggered by each word. Responses were coded for specificity (specific, extended, categoric, or association).	AD patients produced significantly fewer specific memories ($p = .022$) and produced significantly more categoric memories ($p < .0005$) than control subjects.<br>AD patients performed significantly worse on measures of executive function, verbal IQ, spatial IQ, and processing speed (all $p < .01$)<br>In the AD, there was a significant positive correlation between number of categoric memories and semantic fluency ($p < .005$): the greater the semantic fluency, the more categoric memories were generated.

**Müller et al., 2013**	To evaluate AM in different Multiple Sclerosis (MS) subtypes and investigate similarities with patients with AD	20 patients with probable AD<br>Mean age: 73,9 (SD 4.4)<br>Sex: F and M<br><br>20 patients with aMCI<br>Mean age: 72,6 (SD 6.8)<br>Sex: F and M<br><br>32 patients with secondary progressive MS (SPMPS)<br>Mean age: 57.8 (SD 5.7)<br>Sex: F and M<br><br>20 patients with relapsing–remitting MS (RRMS)<br>Mean age: 57.9 (SD 5.2)<br>Sex: F and M<br><br>20 control subjects<br>Mean age: 71.9 (SD 6.5)	AMI [Kopelman et al., 1999]	Recalling personal semantic content and autobiographical incidents from child-hood, early adult life, and recent years	The AD group performed worse than SM and HC in both episodic and semantic memories.<br>AD scores for incident memories from childhood and from early adulthood were poorer for episodic memory.<br>AD scores for semantic memories from early adulthood and recent life were poorer.

		Sex: F and M			
**Müller, S. et al., 2016**	To investigate whether the extent and severity of retrograde amnesia in AD is mediated by the frequency of recall or depends on the age of knowledge.	19 patients with AD Mean age: 72.11 (SD 4.9) Sex: F and M<br><br>20 patients with aMCI Mean age 73.0 years (SD 4.5) Sex: F and M<br><br>21 control subjects Mean age 72.4 years (SD 6.5) Sex: F and M	AMI [Koperlman et al., 1989]	Recalling and describing nine personally experienced life episodes (three from each of three life periods: childhood, early adulthood, and recent life) Responses were scored on a 0-3 point scale based on specificity and detail	Significant main effects of group ($p < 0.001$) and time segment ($p < 0.001$) on AM scores, with AD performed worse than HC in recalling autobiographical incident memories from all life periods ($p = 0.011$ to $p < 0.001$).<br>Significant group × time segment interaction ($p < 0.001$), indicating presence of a temporal gradient, with both AD and aMCI groups showing impaired recall following Ribot's gradient (better preservation of remote vs. recent memories).<br>Significant main effect of retrieval frequency ($p < 0.001$) with frequently retrieved memories recalled in more detail across all groups.
**Philippi et al., 2011**	To investigate brain activity patterns of autobiographical memory loss in AD and control subjects, focusing on the	15 patients with probable AD Mean age: 76,7 (SD 5,62) Sex: F and M<br><br>11 control	MCT [Graham et al., 1996]	Recalling detailed and specific memories in response to 6 cue words, each prompted for the 5 different	AD patients showed significantly lower AM scores compared to HC for all periods except childhood (0-9 years) ($p < 0.05$ for all other periods).AD patients had significantly higher scores for the '10–29 years' period than any other period significant lower scores for 'recent year' compared to any other period ($p= .02$).<br>VBM analysis revealed correlations between AM deficits and atrophy in regions including the hippocampus bilaterally, medial prefrontal cortex, and lateral temporal cortex.<br>Left hippocampal involvement was greater for remote periods, while right hippocampal involvement was greater for more recent periods.

	medial temporal lobe structures.	subjects<br>Mean age: 73,3 (SD 4,80)<br>Sex: F and M		lifetime periods. Memories were scored on a 5-point scale based on specificity and detail.	A rostrocaudal gradient was observed in hippocampal involvement, with anterior regions related to remote memory deficits and posterior regions related to recent memory deficits.
**Philippi et al., 2015**	To examine the influence of emotions on AM in AD patients compared to control subjects, and to study the relationship between emotional autobiographical memory deficits and amygdalar-hippocampal atrophy.	18 patients with probable AD<br>Mean age: 77.17 (SD 6.42)<br>Sex: F and M<br><br>18 control subjects<br>Mean age: 73.67 (SD 5.37)<br>Sex: F and M	Modified Crovitz Test (MCT) [Graham and Hodges, 1997]	Recalling 30 memories in response to 6 cue words, each prompted for 5 life periods	AD showed impairment in recalling of emotional autobiographical memories compared to HC ($p < 0.001$ for scores, $p = 0.002$ for rates)<br>Specificity of emotional memories was preserved in AD compared to their neutral memories and compared to HC. AD patients reported higher mean rates of neutral memories compared to control subjects ($p = 0.002$) but lower mean specificity-scores for this category ($p < 0.001$).<br>Emotional memory deficits in AD correlated with right amygdalar ($p = 0.004$) and hippocampal ($p = 0.003$) volumes.
**Pyo et al., 2011**	To evaluate the effectiveness of the Working Group's Autobiographical Memory Test as a screening	21 AD patients including 10 AD patients with with Down Syndrome	Working Group's Autobiographical Memory Test [(Burt & Aylward, 1998]	Answering three questions: "What is your name?", "When is your birthday?",	At initial assessment, AD scored significantly lower than HC ($p = 0.021$).<br>At 1-year follow-up, no significant difference between AD and HC mainly due to decline in controls patients with DS.<br>Almost all participants correctly answered their name, but most could not give their proper age, regardless of AD status.

	tool for dementia in people with moderate to severe intellectual disabilities (ID).	(DS)<br>Mean age: 48.26 (SD 2.43) and 11 participants with no DS<br>Mean age: 57.99 (SD 11.14)<br><br>42 control subjects including 13 patients with DS<br>Mean age: 47.71 (SD 5.21)<br>and 29 non-DS<br>Mean age: 51.93<br>(SD 7.05)		and "How old are you?", in free recall, multiple choice, and yes/no recognition formats.	
**Ramanan et al., 2021**	To examine AM retrieval across the lifespan in patients with AD, in patients with Logopenic Progressive Aphasia (LPA) and healthy controls.	18 patients with probable AD<br>Mean age: 73 (SD 7.9)<br>Sex: F and M<br><br>10 patients with LPA<br>Mean age:	AI [Levine et al., 2002]	Recalling one specific autobiographical event from each of 4 time periods (teenage years, early adulthood, middle	AD and LPA patients recalled fewer internal episodic details compared to HC (both p<0.005), irrespective on time period. AD and LPA patients produced a lower proportion of internal details within the overall autobiographical narrative compared to HC (both p values <0.001).<br>AD patients produced significantly fewer Event details compared to HC (p < 0.001).

		71.8 (SD 10)<br>Sex: F and M<br><br>16 control subjects<br>Mean age: 74.4 (SD 4.9)<br>Sex: F and M		adulthood, recent past) under free recall and probed recall conditions.	
**Rasmussen et al., 2021**	To investigate how frequently and to what degree patients with AD experience involuntary (spontaneous) autobiographical memories (IAMs)	21 patients with probable AD<br>Mean age: 77.62 years, (SD 6.74)<br>Sex: F and M<br><br>22 control subjects<br>Mean age = 78.14 (SD 5.66)<br>Sex: F and M	IAMs were considered as spontaneous comments with memory content with a personal reference.	Film-only condition: watching a nostalgia film.<br><br>Reminiscence + film condition: recalling life story in a 15-min free narrative and watching a nostalgia film	AD expressed more IAMs overall compared to HC ($p < .05$)<br>AD and HC verbalized more IAMs in the reminiscence+film condition $p < .05$)<br>AD patients spontaneously reported more personal semantics in response to the films than HC.
**Rasmussen et al., 2022**	To examine cultural life script knowledge in	21 patients with probable AD	Standard cultural life-script task (Berntsen &	Envision an infant name and identify the 7 most	AD patients produced significantly fewer life-script events than HC ($p = .001$).<br>Content of events generated by AD was consistent with cultural norms<br>AD showed greater impairment in providing normative timing estimates for events ($p < .001$)

	older adults diagnosed with AD particularly in terms of knowledge for the content of life-script events and the timing and temporal order of these events.	Mean age: 77.62 (SD 6.74)<br>Sex: F and M<br><br>22 control subjects<br>Mean age: 78.14 (SD 5.66)<br>Sex: F and M	Rubin, 2004)	significant milestones expected to occur throughout their lifespan, from birth to death and estimating ages for these events.	AD's life scripts showed lower degree of chronological order (higher proportion of backward pairs, p = .014)<br>AD showed more pronounced positivity bias in life scripts compared to controls (p = .041)
**Rasmussen et al., 2023**	To examine open-ended life stories in patients with AD, focusing on content, narrative coherence, emotional valence, and temporal distribution of life story memories.	21 patients with probable AD<br>Mean age: 77.62 (SD 6.74)<br>Sex: F and M<br><br>22 control subjects<br>Mean age: 78.14 (SD 5.66)<br>Sex: F and M	Life story narratives [Fromholt & Larsen, 1991; Fromholt et al., 2003]<br><br>Coding for event specificity, life script events, emotional valence, facts, and narrative coherence	Recalling the events that have been important in their life for about 15 minutes. Dating of events mentioned in narratives.	AD patients provided significantly fewer life story memories overall compared to controls (p < .001).<br>There was no significant difference in the proportion of specific, extended, or categoric events between AD patients and controls.<br>AD patients included a significantly lower percentage of negative events in their life stories compared to controls (p = .005).<br>AD patients' narratives were significantly less coherent temporally (p < .001) and ended more often in the past rather than the present compared to controls (p < .01).<br>AD patients showed significantly reduced global causal (p < .001) and thematic coherence (p < .001) in their narratives.<br>Both AD patients and controls showed a reminiscence bump, with more memories from young adulthood. However, AD patients had significantly fewer memories from young adulthood (p < .001), adulthood (p < .001), and late adulthood (p = .002) compared to controls.<br>For AD patients, a high proportion of memories within the reminiscence bump period were life script events, similar to controls (no significant difference between groups (p = .407).
**Rathbone et al., 2019**	To compare autobiographical memories	16 patients with AD<br>Mean age:	AMI [Kopelman et	IAM Task: Participants generated	AD generated fewer self-images (p=.001) and self-supporting memories (p=.001) than HC. AD and HC showed similar temporal clustering of memories around self-formation periods.

	cued by self-images and non-self-related cues in AD patients compared to HC considering	68.63 (SD 7.12)<br>Sex: F and M<br><br>29 control subjects<br>Mean age: 70.07 (SD 3.03)<br>Sex: F and M	al., 1989]<br><br>Logical Memory Test (LMT) [Wechler et al., 1997]	self-images ("I am" statements) and memories cued by self-images and non-self-related cues	AD's self-supporting memories were more positive than HC (p=.005)<br>AD and HC reported more category-cued memories when exposed to self-images (AD, p=.02; HC, p=.001).
**Rauchs et al., 2013**	To explore AM for recent events and its relationship with sleep in patients with mild AD	14 patients with probable AD=14;<br>Mean age: 77.1, (SD 4.1)<br>Sex: F and M<br><br>14 control subjects<br>Mean age: 75.1 years, (4.6)<br>Sex: F and M	Adaptation of the TEMPau task [Piolino et al., 2003; 2009]<br><br>Remember/Know paradigm	Recalling personal events from four time periods:<br><br>-Remote (18-30 years old)<br>-Last 2 years (except last month)<br>-Last month<br>-Today and yesterday	AD patients reported lower scores in P2, P3, P4 (all p values < 0.05) than HC but not for P1 (p > 0.39).<br>AD showed relatively better recall for very recent events (today/yesterday) compared to intermediate periods (p < 0.002).<br>The number of justified Remember responses for events experienced before sleep positively correlated with the amount of slow-wave sleep in AD (r = 0.60, p < 0.05).<br>AD provided more justified Remember responses for events from the previous day compared to same-day events (p = 0.007).<br>Recall of recent events correlated with glucose metabolism in several brain regions including the precuneus and retrosplenial cortex (p < 0.001).
**Rodrigues, G. R. et al., 2015**	To translate, make cross-cultural	11 patients with AD<br>Mean Age:	EAMI [Irish et al., 2008]	Recalling memories across the	EAMI scores were significantly lower in AD compared to HC (p < 0.001)<br>Strong correlation between EAMI and delayed recall of MFT (p < 0.001)<br>Strong correlation between EAMI and Remember-Know coefficient (p < 0.001)

	adaptation and validate the Autobiographic Episodic Memory Interview (EAMI) for use in a Brazilian population.	76.3 years (SD 6.98)<br><br>10 control subjects Mean age: 71.3 (SD 4.24)	Memory of Figures Test (MFT) Remember-Know paradigm	lifespan. It includes sections on Personal Semantics, Autobiographical Event Recall, and Autonoetic Awareness Assessment.	High inter-rater reliability (intraclass correlation coefficient 0.98-0.99)<br>Good internal consistency (Cronbach's α 0.73-0.86)
**Sadek et al., 2004**	To investigate retrograde amnesia in patients with AD and Huntington's disease (HD) and in individuals diagnosed with HIV-associated dementia (HIV-D).	11 patients with probable AD Mean age: 74.7 (SD 6.0) Sex: F and M<br><br>7 patients with HIV-D Mean age: 38.0 (SD 8.7) Sex: M<br><br>12 patients with HD Mean age: 46.8 (SD 13.2) Sex: F and M<br><br>17 younger control subjects	Remote Memory Battery, [Albeter al., 1981] which includes:<br>-Famous Faces Test<br>-Public Events Questionnaire	Recalling information from different decades (1940s through 1990s) recording free recall, semantic cues, phonemic cues.	AD patients performed worse than all other groups ($p< .001$) and performed worse for recent decades than for more remote decades (1940s vs. 1980s, p .03; 1950s vs. 1990s, p .04).<br><br>AD patients showed a distinct temporal gradient compared to other groups (HIV-D and HD) reporting more impaired recall of information from recent decades (p .001 in all instances)and relatively preserved recall of information from distant decades (p .001 in all instances)

		Mean age: 40.0 (SD 7.5)<br>Sex: F and M<br><br>23 older control subjects<br>Mean age: 71.4 (SD 7.0)<br>Sex: F and M			
**Sartori et al., 2004**	To investigate AM and knowledge for public events in advanced AD and control subjects.	10 patients with probable AD<br>Mean age: 83.1 (SD 9.0)<br>Sex: F and M<br><br>10 control subjects<br>Mean age: 83.1 (SD 9.0)<br>Sex: F and M	The Autobiographical Memory Enquiry (AME) [Borrin et al., 1989]<br><br>Autobiographical fluency (AF) [Dritschel, Williams, Baddeley, et al. 1992]	AME=Answering five questions regarding particular autobiographical events for three life periods (childhood, early adulthood, late adulthood)<br><br>AF=Producing names and incidents from specified life periods within 90 seconds	AD were impaired on all measures of remote memory compared to HC ($p < .05$ for all measures except AF names, which was borderline significant at $p = .054$).<br>Both AD and HC showed better preserved memory for earlier public events compared to more recent events (Time Period effect: $p = .007$).<br>Semantic fluency was significantly correlated with autobiographical and remote public event memory performance in AD only (correlations ranged from $r = .57$ to $r = .72$, all $p < .05$).

**Seidl et al., 2011**	To investigate semantic and episodic aspects of AM for remote and recent life periods in nursing home residents with AD and MCI and in a group of HC.	165 patients with probable AD<br>Mean age for Mild AD: 84.3 (SD 7.8),<br>Mean age for moderate AD: 86.9 (SD 6.1)<br>Mean age for severe AD: 87.1 (SD 7.0)<br>Sex: F and M<br><br>33 patients with MCI<br>Mean age: 79.3 (SD 6.9)<br>Sex: F and M<br><br>41 healthy control subjects<br>Mean age: 76.0 (SD 4.7)<br>Sex: F and M	Extended Autobiographical Memory Inventory (E-AGI) [Fast et al., 2007]	Recalling personal episodes that were rated according to the uniqueness of narrative statements and to the richness of details.	AM was impaired even in early stages of AD and declined further with disease progression ($p < 0.001$).<br>Semantic AM remained stable in mild AD but deteriorated in moderate and severe stages ($p < 0.001$).<br>Episodic AM (both free recall and richness of details) was compromised early, even in MCI ($p < 0.001$).<br>Moderate AD patients showed reduced performance for recent 5 years compared to other periods ($p < 0.01$).
**Starkstein et al., 2005**	To investigate long-term changes in autobiographical and public remote memory in AD over a 2-3 year	17 patients with probable AD<br>Mean age: 74.5 (SD 6.2)<br>Sex: F and M<br><br>10 control	Autobiographical Memory Scale<br>Remote Memory Scale [Dorrego et al., 1999]	Answering 39 questions assessing 5 life periods (childhood, adolescence, youth, adulthood,	AD showed significantly greater decline in both AM and public remote memory compared to HC ($p<0.05$ for AM, $p<0.01$ for public memory).<br>AD performed significantly better on recognition than free recall for both AM and remote memory (Task effect, $p<0.01$).<br>AD showed better recall for childhood/adolescence compared to later periods ($p<0.01$).<br>AD reported a temporal gradient (better recall of earlier decades) at follow-up for public memory in AD $p<0.05$).

	period.	subjects<br>Mean age 72.1 (SD 9.8)<br>Sex: F and M		recent events) based on free recall and recognition	Decline in anterograde verbal memory correlated significantly with decline in AM (p<0.01).
**Strikwerda-Brown et al., 2022**	To examine the nature of "external" details provided during future thinking narratives in patients with AD and SD, using a fine-grained scoring system called NExt (New External Details Taxonomy).	11 patients with probable AD<br>Mean age: 64.64 (SD 6.10)<br>Sex: F and M<br><br>13 patients with SD<br>Mean age: 63.00 (SD 5.31)<br>Sex: F and M<br><br>15 control subjects<br>Mean age: 69.27 (7.10)<br>Sex: F and M	Past-Future task [Addis et al., 2008]<br><br>NExt taxonomy [Strikwerda-Brown et al., 2018]	Describing in detail specific, personally relevant events that had occurred within the past 12 months or that might occur in the next 12 months, in response to cue words accompanied by visual images.	AD patients provided significantly more Specific Episode external details compared to HC (p = .009).<br>SD patients reported more Specific Episode details (p = .002), more Extended Episode details (p = .002) and General Semantic details (p = .008) during future simulation relative to HC.<br>In AD, increased Specific Episode details correlated with grey matter intensity in medial and lateral frontal regions.<br>In SD, external detail increases correlated with grey matter intensity in medial and lateral parietal regions.
**Westmacott et al, 2004**	To investigate the relationship between personal experiences and general	18 patients with probable AD<br>Mean age 75.47 (SD 6.50)	Remember/Know task [Knowlton, 1998; Markowitsch et al.,1997]	To make Remember/Know judgments about famous names, indicating if	AD patents exhibited significantly poorer performance in tasks involving recognition and identification compared to the control subjects.<br>AD patients provided significantly fewer "Remember" responses compared to control subjects.

	knowledge by evaluating patients with neurological conditions, with impaired autobiographical memory and others who did not.	Sex: F and M<br><br>2 patients with SD aged 63 and 64<br><br>4 amnesic patients aged 47-70<br>Sex: F and M<br><br>20 control subjects aged 45-55<br>Mean age: 51.2<br>Sex: F and M<br><br>20 control subjects 20 aged 65-80<br>Mean age 72.6<br>Sex: F and M		they could recollect a specific autobiographical episode associated with the name (Remember) or if they just knew facts about the person (Know).	
